# Regulation of ADAM10 activity through microdomain-dependent intracellular calcium changes

**DOI:** 10.1186/s12964-024-01891-5

**Published:** 2024-11-04

**Authors:** Federico Guillermo Gharzia, Ahmad Aljohmani, Andreas Beck, Stephan E. Philipp, Daniela Yildiz

**Affiliations:** 1 Molecular Pharmacology, PZMS, Saarland University, Campus Homburg Building 46, 66421 Homburg, Germany; 2 Institute of Experimental and Clinical Pharmacology and Toxicology, PZMS, Saarland University, Homburg, Germany

**Keywords:** Calcium, Proteolysis, Metalloproteinases, Exosomes, Transient receptor potential channels, Junction and adhesion molecules

## Abstract

A disintegrin and metalloproteinases (ADAMs) are transmembrane proteases that cleave other proteins close to the surface in a process called *shedding*. The prominent member ADAM10 has been linked to several pathologies such as Alzheimer’s disease, bacterial infection, cancer development and metastasis. Although the regulation of the ADAM10 activity by calcium influx and calmodulin inhibition has been reported, the spatiotemporal regulation of Ca^2+^-dependent ADAM10 activation and the required source of Ca^2+^ ions have not been thoroughly studied. In the present study, we observed the rapid Ca^2+^-dependent activation of ADAM10 in A549 lung carcinoma cells upon stimulation with ionomycin. The calmodulin-inhibitors trifluoperazine and ophiobolin A mediated delayed activation of ADAM10, which apparently did not depend on intracellular Ca^2+^ in the case of trifluoperazine. Furthermore, the surface translocation and release of ADAM10 in extracellular vesicles exhibited different kinetics and were only partially linked to catalytic activation. Finally, ADAM10 activation was observed after the entry of Ca^2+^ through certain channels, such as canonical members of transient receptor potential (TRP) channels. Therefore, the opening of particular channels for Ca^2+^ entry points and subsequent Ca^2+^ flux as well as the temporal aspects of the consequent increase in Ca^2+^ levels, must be considered for future therapeutic options involving the increasing or decreasing ADAM10 activity.

**Supplementary Information:**

The online version contains supplementary material available at 10.1186/s12964-024-01891-5.

## Background

A disintegrin and metalloproteinases (ADAMs) are transmembrane multidomain proteases that cleave other proteins close to the cell surface in a process called ectodomain shedding [1]. ADAM10 has been linked to Alzheimer’s disease due to its cleavage of the amyloid precursor (AP) protein, which generates an α-fragment and prevents the generation and aggregation of APβ fragments, thus limiting disease progression [2]. Furthermore, its activity plays a central role in Notch-dependent developmental processes, especially in the brain cortex and in vessel formation during embryogenesis [3]. Additionally, E-cadherin, which is mainly responsible for the formation of adherens junctions, has been reported to be a substrate of ADAM10 [4]. E-cadherin cleavage is linked to pathophysiological events in lung infection [5] and cancer metastasis since it promotes the epithelial-to-mesenchymal transition [6].

ADAM10 activity has been shown to be induced in a Ca^2+^-dependent manner. However, these studies focused on Ca^2+^ entry through the activation of scramblases or the detachment of calmodulin from the C-terminal tail [7, 8]. Nonexcitable cells possess many of potential proteins and strategies for Ca^2+^ signaling, such as the activation of cationic transient receptor potential (TRP) channels. Although all family members share the 6-transmembrane domain structure, they show different homologies and a broad diversity of cation selectivities [9]. A recent study reported that the cleavage of EGFR ligands by metalloproteases is a feasible readout for the activation of TRP channels [10]. ADAMs not only are regulated at the cell-associated level but might be released as soluble ectodomains or as extracellular vesicle (EV) cargos [11, 12]. Although the release of EVs, including microvesicles (> 100 nm) and exosomes (10–100 nm), is generally considered Ca^2+^-dependent [13, 14], this phenomenon has not been studied in detail for ADAMs and their substrates.

The present study aimed to further elucidate the spatiotemporal regulation of Ca^2+^ -dependent ADAM10 activation, focusing on the Ca^2+^ source, concentration, and time needed for the activation of ADAM10. We describe for the first time the time- and threshold-dependent activation of ADAM10 by intracellular Ca^2+^ transients. We observed the rapid Ca^2+^-dependent activation of ADAM10 upon stimulation with ionomycin which was delayed upon treatment with trifluoperazine (TFP) or ophiobolin A (OphA). On the other hand, the translocation and release of ADAM10 in soluble form or in exosomes occurred faster when cells were stimulated with TFP. In addition, ADAM10 was activated by Ca^2+^ influx through specific entry points such as canonical TRP channels, including TRPC4 and TRPC5. Global broad-spectrum inhibition or activation of ADAM10 as a therapeutic option is not feasible due to severe side-effects. However, this novel understanding of the spatiotemporal control and microdomain-dependent activation of ADAM10 offers a great potential for future translational studies to develop novel therapeutic options.

## Materials and methods

### Antibodies, reagents, buffers, and solutions

The antibodies, special reagents, buffers and solutions used are listed in Tables 1 and 2.

**Table 1:** Antibodies and reagents

Reagent	Company	Working Concentration
Ionomycin	Merck Millipore (Darmstadt, Germany)	10 µM – 0.1 µM
Trifluoperazine	Merck Millipore (Darmstadt, Germany)	100 µM
Ophiobolin A	Merck Millipore (Darmstadt, Germany)	10 µM
Thapsigargin	Invitrogen by Thermo Fisher Scientific (Frankfurt, Germany)	1 µM
GI254023X	Merck Millipore (Darmstadt, Germany)	10 µM
BAPTA-AM	Invitrogen by Thermo Fisher Scientific (Frankfurt, Germany)	10 µM
EGTA	Merck Millipore (Darmstadt, Germany)	3 mM
Fura2-AM	Invitrogen by Thermo Fisher Scientific (Frankfurt, Germany)	5 µM
Monoclonal E-cadherin C-terminal Antibody	BD Biosciences (Heidelberg, Germany)	0.25 µg/ml
Monoclonal E-cadherin N-terminal Antibody	Santa Cruz Biotechnology (Dallas, Texas, USA)	0.2 µg/ml
Polyclonal ADAM10 C-terminal Antibody	Invitrogen by Thermo Fisher Scientific (Frankfurt, Germany)	1 µg/ml
ADAM10 N-terminal Antibody	R&D System (Wiesbaden, Germany)	1 µg/ml
TRPC4 Antibody	Affinity-purified	0.5 µg/ml
TRPC5 Antibody	Affinity-purified	0.5 µg/ml
TRPM3 Antibody	Affinity-purified	1 µg/mL
Anti-mouse A-21,235 Secondary Antibody	Invitrogen by Thermo Fisher Scientific (Frankfurt, Germany)	8 µg/ml
Monoclonal Anti-mouse IgG2B Isotype Control Antibody	R&D System (Wiesbaden, Germany)	1 µg/mL

**Table 2:** Buffer and solutions

Buffer/Solution	Composition
PBS	136 mM NaCl, 2.7 mM KCl, 1.8 mMKH_2_PO_4_, 10 mM Na_2_HPO_4_
ADAM10 lysis buffer	5 mM Tris-HCl, 1 mM EGTA, 250 mM Saccharose, 0,1% (w/v) SDS 1% TritonX-100, 1x Complete EDTA-free Protease Inhibitor cocktail (Merck Millipore (Darmstadt, Germany)), 1 mM Na_3_VO_4_, 1 mM PMSF, 10 mM 1,10-phenantroline
IP lysis buffer	1% Brij97, 10 mM Tris pH 7.5, 150 mM NaCl, 2 mM CaCl_2_, 2 mM MgCl_2_, 0.02% sodium azide, 1x complete
SDS buffer	250 mM Tris HCl (pH 6.8), 50% glycerol, 10% SDS, 0.1% bromophenol blue, 5% β-mercaptoethanol
FACS buffer	136 mM NaCl, 2.7 mM KCl, 1.8 mM KH_2_PO_4_, 10mM Na_2_HPO_4_, 0.2% BSA
Alkaline phosphatase assay lysis buffer	1% Triton-X100, 50 mM Tris, 0.15 M NaCl, 1x Complete; pH 7,5
Alkaline phosphatase assay activity buffer	40 mM Tris-HCl, 40 mM NaCl, 10 mM MgCl_2_, pH 9.5
Ringer’s solution	140 mM NaCl, 4 mM KCl, 1 mM MgCl_2_, 10 mM HEPES, 10 mM D-glucose
ADAM10 FRET-based activity assay buffer	20 mM Tris, 0,0006% Brij-35, pH 8.0
0Ca/EGTA saline solution	140 mM NaCl, 4 mM KCl, 1 mM MgCl_2_, 10 mM HEPES, 10 mM D-glucose, 10 mM EGTA
10Ca/EGTA saline solution	140 mM NaCl, 4 mM KCl, 1 mM MgCl_2_, 10 mM HEPES, 10 mM D-glucose, 10 mM EGTA, 10 mM CaCl_2_, 20 µM 4Br-A23187
20Ca saline solution	140 mM NaCl, 4 mM KCl, 1 mM MgCl_2_, 10 mM HEPES 10 mM D-glucose; 20 mM CaCl_2_, 20 µM 4Br-A23187

### Cell culture and cell stimulation

A549 and A431 cells were cultured in Dulbecco’s modified Eagle’s medium (DMEM) supplemented with 10% fetal calf serum (FCS), and HEK293 cells were cultured in minimum essential medium (MEM) supplemented with 10% FCS. HEK-µOR TRPC4α [15] cells were cultured in DMEM supplemented with 10% FCS, 100 µg/mL hygromicin B, and 500 µg/mL G418. HEK-TRPM3α2 [16] and HEK-CCE2 [17] (TRPC5) cells were cultured in MEM supplemented with 10% FCS and G418 (500 and 400 µg/mL, respectively). All the cells were incubated at 37 °C in a 5% CO_2_ atmosphere. For stimulation experiments, cells were seeded in 6-well plates and grown until they reached confluence. The cells were starved with 0.5% FCS media overnight, followed by an incubation in FCS-free media for 1 h and stimulation as indicated in the figure legends. For ADAM10 inhibition, a preincubation step with 10 µM GI254023X for 30 min was included prior to stimulation. The cells were subsequently lysed with ADAM10 lysis buffer, followed by centrifugation for 15 min at 16,000 x g and storage of the supernatant at -80 °C.

### Western blot analysis and E-cadherin cleavage calculation

The protein concentration of the cell lysates was quantified using a bicinchoninic acid (BCA) assay kit (Thermo Fisher, Karlsruhe, Germany) according to the manufacturer’s protocol. The samples were heated in SDS buffer at 60 °C for 30 min before being subjected to SDS‒PAGE (10% Tris‒glycine gels), in which equal protein amounts were loaded per lane and blotting onto nitrocellulose membranes (Amersham Protran Premium 0.45 NC, GE Healthcare Life Sciences, Freiburg, Germany). The membranes were blocked with 5% nonfat milk in Tris-buffered saline containing 0.05% Tween for 1 h, incubated with the primary antibody overnight (Table 1) and then incubated with the corresponding secondary antibody for 1 h at RT. The chemiluminescence substrate (PerkinElmer, Waltham, MA, USA) was added to the membranes, and the chemiluminescent signals were recorded using a LAS3000 device (Fujifilm, Tokyo, Japan). AIDA Image Analysis software 4.27.039 (Elysia-raytest, Angleur, Belgium) was used for image acquisition and densitometric analysis after background subtraction utilizing equal square sizes for the bands of interest. For the determination of E-cadherin cleavage activity, membranes were developed using an anti-mouse C-terminal E-cadherin antibody, and the densitometry analysis was performed by calculating the ratio of the 36 kDa C-terminal fragment (CTF) to the 120 kDa full-length (FL) protein with the following formula: $\:\frac{Intensity\:CTF}{Intensity\:FL}$. The results were normalized to the amount of protein loaded onto the gels.

### Coimmunoprecipitation

HEK-µOR TRPC4, HEK-TRPM3α2 and HEK-CCE2 cells were lysed in IP lysis buffer and equilibrated at 4 °C for 30 min. Subsequently, the lysates were centrifuged for 45 min at 100,000 x g, and the supernatants were incubated with antibody-coupled beads overnight at 4 °C. For the preparation of the antibody-coupled beads, 60 µL of Protein G Dynabeads (beads) per sample was washed twice with IP lysis buffer (magnetic stand). The appropiate amount of beads was incubated with 10 µg of TRPC4, TRPC5, TRPM3 or ADAM10 C-terminal antibody in of 500 µL IP lysis bufferfor 1 h, and washed five times with IP lysis buffer to remove the unbound antibody. After the overnight incubation of the beads and lysates, the beads were collected (magnetic stand) and denatured in SDS buffer for 20 min at 60 °C. The samples were then subjected to Western blot analysis developing with antibodies against TRPC4, TRPC5, TRPM3 or the ADAM10 C-terminus as described above.

### Antibody surface staining and flow cytometry

A549 and A431 cells were dissociated with accutase (Merck Millipore, Darmstadt, Germany), resolved in FACS buffer and centrifuged for 5 min at 300 x g. The cells were kept on ice throughout the following steps. The supernatant was removed, and the cells were incubated with an ADAM10 MAB004 antibody (1 µg/mL), isotype control (1 µg/mL) or FACS buffer for 1 h. The specificity of the ADAM10 antibody was reported in a previous publication with ADAM10 deficient cells [18]. The cells were washed twice and incubated with the secondary anti-mouse antibody A-21235; (2 µg/mL) for 45 min. Afterwards, the cells were washed twice, fixed in 1% paraformaldehyde (PFA) for 5 min and finally diluted with 0.1% PFA for fluorescence signal detection using the Sony SH800 (Sony, Berlin, Germany) and FACS Aria Fusion, BD, Heidelberg, Germany) systems. An equal amount of cells was recorded per sample. The data were analyzed with FlowJo 10.6.2 software (Tree Star, Inc., Ashland, OR, USA). Cells with decreased viability and cell debris were excluded by forward and side scatter gating, and the mean fluorescence of the samples minus the mean fluorescence of the isotype control was used for comparison.

### Alkaline phosphatase (AP) assay

A549 cells were transfected with a plasmid encoding N-terminally coupled alkaline phosphatase (AP)-betacellulin fusion protein using Lipofectamine™ 3000 (Thermo Fisher, Karlsruhe, Germany) and seeded in 12-well plates at a density of 3*10^5^ cells per well [19]. After the respective treatments and sampling, 100 µl of the cell culture supernatant and the corresponding cell lysate (in AP-assay lysis buffer) was transferred to a 96-well plate, and 100 µl of a 2 mg/ml 4-nitrophenyl phosphate solution (AP substrate, resolved in AP-assay activity buffer) was added to each well. Importantly, the plate must be kept cool/on ice before the start of the measurement to avoid unintended substrate turnover. Protease activity was quantified as equivalent substrate turnover by AP by measuring of the absorption at 405 nm every 1.5 min for 2.5 h at 37 °C using Genios fluorescence reader (Tecan, Grödig, Austria). Finally, the slopes of the linear region of the curve were determined, and the ratio of the activity in the supernatant to the activity in the lysate plus the supernatant (total activity) was calculated to obtain the final activity in the sample.

### Exosome preparation

A total of 2 × 10^7^ A549 cells were seeded in 100 mm dishes, and after adherence, the cells were cultured overnight in DMEM containing 0.5% FCS. The cells were further starved with TBS with or without Ca^2+^ followed by ionomycin or trifluoperazine stimulation. The medium was collected and subjected to differential centrifugation (10 min at 300 x g, 20 min at 1,000 x g, 30 min at 10,000 x g). After each centrifugation step, the pellets were lysed with SDS buffer, and the supernatants were subjected to the next centrifugation step. The supernatants were filtered through a 0.22 μm membrane filter, and the extracellular vesicles were collected by centrifugation at 100,000 x g for 1 h at 4 °C using a Beckman rotor Type Ti50.2. The pellet was washed with ice-cold PBS and then centrifuged at 100,000 x g for 1 h. Vesicles were directly lysed with SDS-sample buffer for Western blot analysis or further fractionated by gradient centrifugation for pure exosome preparation as described previously [12]. Briefly, the vesicle pellets were resuspended in PBS and loaded onto a continuous sucrose gradient (2, 1.3, 1.16, 0.8, 0.5 and 0.25 M sucrose). After centrifugation at 100,000 x g for 16 h at 4 °C (Beckman Ti50.2 rotor), six 1 ml fractions were collected, and the density of each fraction was measured and subsequently centrifuged at 150,000 x g for 4 h at 4 °C using a Beckman TLA-55 rotor. The pellets were collected and subjected to Western blot analysis.

### Measurement of cytoplasmic Ca^2+^ levels with Fura-2

A549 cells (2.5*10^5^) were seeded on poly-L-lysine (Sigma, Steinheim, Germany) coated glass coverslips and grown until 70% confluence. The cells were loaded with 4 µM Fura-2 AM (Invitrogen by Thermo Fisher Scientific, Eugene, Oregon, USA) for 30 min at 37 °C and then washed with Ringer’s solution with or without Ca^2+^. The coverslips were placed into a circular open-bottom chamber, and washed again before the chamber was fitted onto the stage of an inverted microscope (Axivert S100, Carl Zeiss, Jena, Germany) with a monochromator (Polychrome V, TILL Photonics, Germany). The measurements were performed at room temperature. Every 2 s, the fluorescence from Fura-2 was captured by alternating excitation (0.5 Hz) at 340 and 380 nm for 20 ms, while the emission was recorded with a cooled charge-coupled device (CCD) camera (Imago, TILL Photonics). The cells were marked as regions of interest (ROIs) whereas two to three empty areas were selected as backgrounds to be subtracted. The changes in the ratios of single ROIs were recorded, and the F340/380 ratios versus time were plotted automatically. Individual cells with significant deviations were considered outliers based on the observations of the XY ratio. Monochromator operator, image acquisition, and analysis were controlled by TillvisION software (TILL Photonics, Planegg/Martinsried, Germany). The final results are shown as the F340/F380 ratio, area under the curve or amplitude.

### Fura-2 AM calibration and Ca^2+^ concentration calculation

The cells were seeded and treated as described above. After the washing steps, Ringer’s solution was removed and replaced with 160 µL of 0Ca/EGTA solution, and the imaging experiment for the cytoplasmic Ca^2+^ measurement was started. When the signal plateau was reached, the Rmin (F340/380 value) and Sf2 (excitation intensity of the 380 nm channel in Ca^2+^ free conditions) values were extracted. A total of 240 µL of 10Ca/EGTA solution was added resulting in a final Ca^2+^ concentration of 110 nM (calculated using the webmaxc standard). In the plateau phase, the R110nM/Ca value was extracted. Finally, 400 µL of 20Ca saline solution was added, and Rmax and Sb2 (excitation intensity of the 380 nm channel in excess Ca^2+^ conditions) values were extracted. Five independent measurements with a total of 170 cells were performed The following equation was used to calculate the experimental dissociation constant:

$$\:{Kd}_{eff}=110nM/\left(\frac{{R}_{110nM}-{R}_{min}}{{R}_{max}-{R}_{260nM}}*\left(\frac{Sf2}{Sb2}\right)\right)$$

The Ca^2+^ concentrations from other Ca^2+^ imaging experiments were calculated using the following equation

$$\:\left[{Ca}^{2+}\right]i={Kd}_{eff}*\frac{{R}_{exp}-{R}_{\text{m}\text{i}\text{n}}}{{R}_{max}-{R}_{exp}}*\left(\frac{Sf2}{Sb2}\right)$$

as described earlier with the dissociation constant value [20].

### sADAM10/exosome related ADAM10 FRET-based activity assay

Soluble or exosome related ADAM10 activity in the supernatant was assessed with the FRET substrate PEPDab063A (Dabcyl-HGDQMAQKSK(5FAM)-NH2) (Biozyme Inc., North Carolina, USA). This substrate contains a consensus cleavage sequence with high specificity for ADAM10 [21]. Upon cleavage, the quencher is released from the fluorophore resulting in an increase in fluorescence. A549 and A431 cells were seeded in 12 well plates, starved overnight in DMEM supplemented with 0.5% FCS, and finally equilibrated in phenol red-free and serum-free DMEM for one hour. After the indicated treatments and sampling, the supernatant was centrifuged, diluted 1:2 in phenol red-free DMEM (50 µL end volume), and added to a 96 well reading plate in triplicate together with a positive control (trypsin) and a negative control containing a 1:1 mixture of ADAM10 FRET-based activity assay buffer and phenol red-free DMEM. The GI inhibitor was directly added to the plates. Importantly, the plate must be kept cool/on ice to avoid unintended substrate cleavage. The FRET-substrate was added to a final concentration of 10 µM, and fluorescence was measured in a TECAN plate reader (Zürich, Switzerland) every 2 min for 4 h with a gain optimization function (excitation wavelength: 485 nm; emission wavelength: 530 nm). For data acquisition, the average of the technical replicates was obtained, and the slope of the linear region of the curve was calculated for each sample. The results were normalized to the experimental control.

### Statistical analysis

The quantitative data are shown as the means + SD from three independent experiments, unless indicated otherwise. The statistical analysis was performed using GraphPad PRISM 9.0 (GraphPad Software, La Jolla, CA). A p-value < 0.05 was considered significant. The statistical analysis of the nonnormalized E-cadherin cleavage data was performed using ANOVA followed by a Tukey’s post- hoc test for multiple comparisons between groups. Significant differences in AP-BTC cleavage (normalized data) were analyzed with a one-sample t-test followed by a false discovery rate (FDR) analysis. For details on the other experiments see the figure legends.

## Results

### Extracellular calcium influx promotes E-cadherin cleavage through activation of ADAM10

The following stimuli were compared in A549 cells to determine the relevance of extracellular Ca^2+^ influx, the release of Ca^2+^ from internal stores, and calmodulin (CaM) for the proteolytic cleavage of E-cadherin: ionomycin an unspecific calcium ionophore for the stimulation of extracellular Ca^2+^ influx; thapsigargin for a molecule that increases the intracellular Ca^2+^ concentration through SERCA blockage and the induction of Ca2^+^ release from the ER; and trifluoperazine (TFP), a CaM-inhibitor that blocks the CaM-dependent inhibition of ADAM10 and Ca^2+^/CaM-dependent feedback inhibition of the IP3 receptor (IP3R) and the RyR receptor [22, 23].

**Fig. 1:**
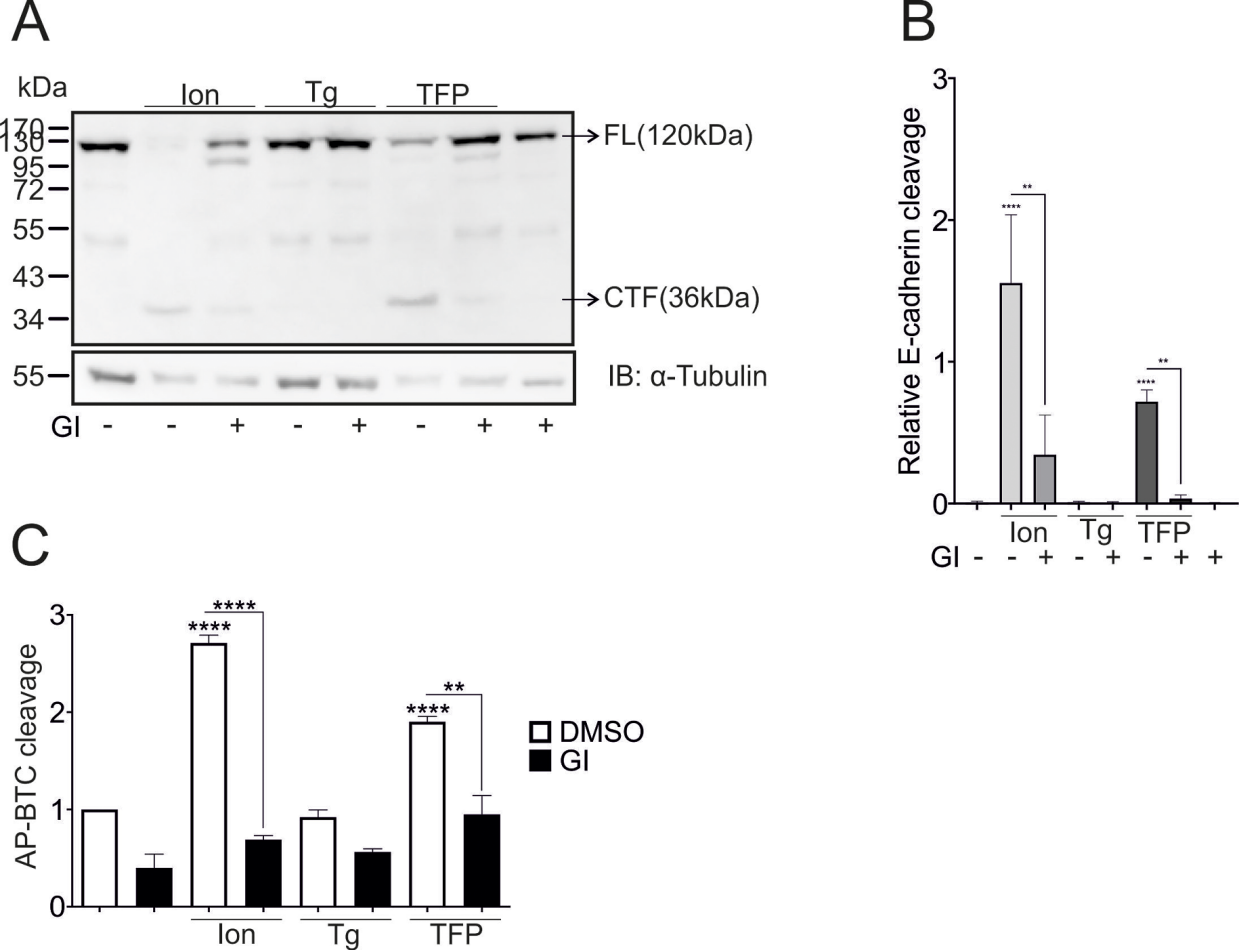
Calcium- and ADAM10-dependent cleavage of E-cadherin. **A**-**C**: Confluent A549 monolayers were starved overnight and stimulated with 10 µM ionomycin, 1 µM thapsigargin, or 100 µM trifluoperazine (TFP) in the absence or presence of 10 µM GI254023X (GI), an ADAM10 inhibitor for 1 h. DMSO (0.1%) served as a vehicle control. Lysates were collected and subjected to E-cadherin cleavage analyses in A and B (*n* = 4). In C, cells were transfected with AP-BTC prior to seeding, and the cleavage of alkaline phosphatase (AP)-conjugated betacellulin (BTC) was analyzed via an alkaline phosphatase (AP) assay (*n* = 3). The quantitative data are shown as the means + SDs. Asterisks without lines represent differences compared with the control, and those with lines represent differences between groups (* *p* < 0.05, ** *p* < 0.01, *** *p* < 0.001 and **** *p* < 0.0001)

Both 10 µM ionomycin and 100 µM TFP induced the cleavage of E-cadherin, an endogenously expressed substrate, as indicated by the generation of the C-terminal fragment (CTF), which could be almost completely abolished by treatment with GI254023X (GI), an ADAM10-specific inhibitor [24]. In contrast, stimulation with thapsigargin had no effect (Fig. 1A, B). These experiments were repeated in A549 cells overexpressing AP-conjugated betacellulin (AP-BTC) to assess whether this result might be a substrate-specific effect, and similar results were obtained (Fig. 1C).

**Fig. 2:**
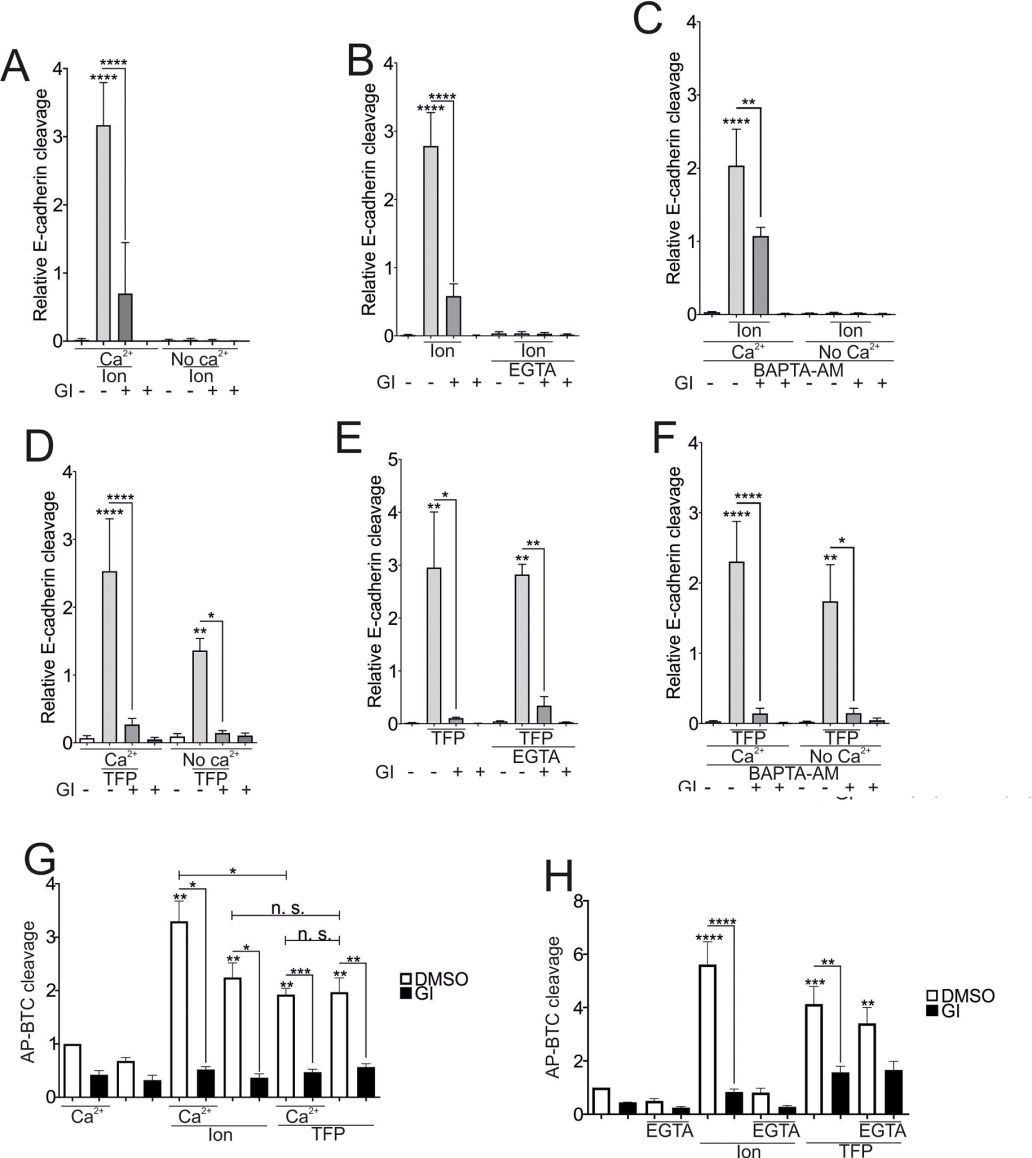
Dependence of ADAM10-mediated E-cadherin cleavage on the origin of the increase in calcium concentration. **A**-**F**: Confluent A549 monolayers were starved overnight and stimulated with 10 µM Ion or 100 µM TFP in the presence or absence of 10 µM GI for 1 h. DMSO (0.1%) served as a vehicle control. Experiments were performed in the presence (2 mM CaCl_2_) or nominal absence of calcium (**A**, **C**, **F**, **G**), with extracellular Ca^2+^ chelation by 3 mM EGTA (noncell permeable, added 30 min before stimulation) (**B**, **E**, **H**) or intracellular Ca^2+^ depletion with 10 µM BAPTA-AM (cell loading 30 min prior to stimulation) (**C**, **F**). Lysates were collected and subjected to E-cadherin cleavage analyses in A to F (*n* = 3). (*n* = 4). In G and H, cells were transfected with AP-BTC prior to seeding, and AP-BTC cleavage was analyzed via the AP assay (*n* = 3). The quantitative data are shown as the means + SDs. Asterisks without lines represent differences compared with the control, and those with lines represent differences between groups (* *p* < 0.05, ** *p* < 0.01, *** *p* < 0.001 and **** *p* < 0.0001)

Experiments conducted in the nominal absence of extracellular Ca^2+^ (containing only the ions remaining after ion exchange purification of used water) or upon depletion of extracellular Ca^2+^ by EGTA revealed that an influx of extracellular Ca^2+^ was required the ionomycin-induced E-cadherin cleavage (Fig. 2A and B - Supplemental figure (SFig. 1 A, B). This finding was further supported by the lack of a significant effect of the BAPTA-AM preincubation (Fig. 2C - SFig. 1 C). BAPTA-AM is a cell-permeable analog of BAPTA that binds calcium only after the acetoxymethyl group is removed by cytoplasmic esterases, thus chelating intracellular Ca^2+^ only. In contrast, cleavage induced by the CaM-inhibitor TFP occurred independently of extracellular Ca^2+^ (Fig. 2D, E;, Fig. 2 - SFig. 1D, E), with no difference in cleavage observed after intracellular Ca^2+^ chelation (Fig. 2F; SFig. 1 F). In all cases, the ADAM10-dependency could be confirmed by the inhibition of E-cadherin cleavage through GI (Fig. 2A-F). For the exogenous substrate AP-BTC, a different result was observed. TFP-induced cleavage was not affected by extracellular Ca^2+^ (Fig. 2G, H). In contrast to E-cadherin cleavage, ionomycin-induced AP-BTC cleavage occurred in the nominal absence of extracellular Ca^2+^, although to a lesser extent than the presence of Ca^2+^ (Fig. 2G). However, the chelation of extracellular Ca^2+^ by EGTA abolished this cleavage (Fig. 2H), whereas BAPTA-AM caused only a nonsignificant reduction in AP-BTC cleavage (Fig. 2 – SFig. 1G). One potential explanation could be the greater sensitivity of betacellulin cleavage to residual calcium under nominal Ca^2+^-free conditions. Therefore, experiments were conducted in the presence of increasing concentrations of extracellular Ca^2+^. Ionomycin-induced E-cadherin-cleavage was prominent in the presence of 1 mM extracellular Ca^2+^ (Fig. 2 - SFig. 2A-D), while significant AP-BTC cleavage was observed in the presence of 0.03 mM extracellular Ca^2+^ (Fig. 2 - SFig. 2E). Thus, ADAM10-mediated cleavage of E-cadherin and betacellulin may depend on extracellular calcium influx with different Ca^2+^ sensitivities, whereas the cleavage induced by TFP stimulation seems to be mainly Ca^2+^-independent.

### Ca2+-induced ADAM10 activation is time- and threshold-dependent

Next, we analyzed the temporal kinetics of the observed Ca^2+^-induced proteolysis mediated by ADAM10.

**Fig. 3:**
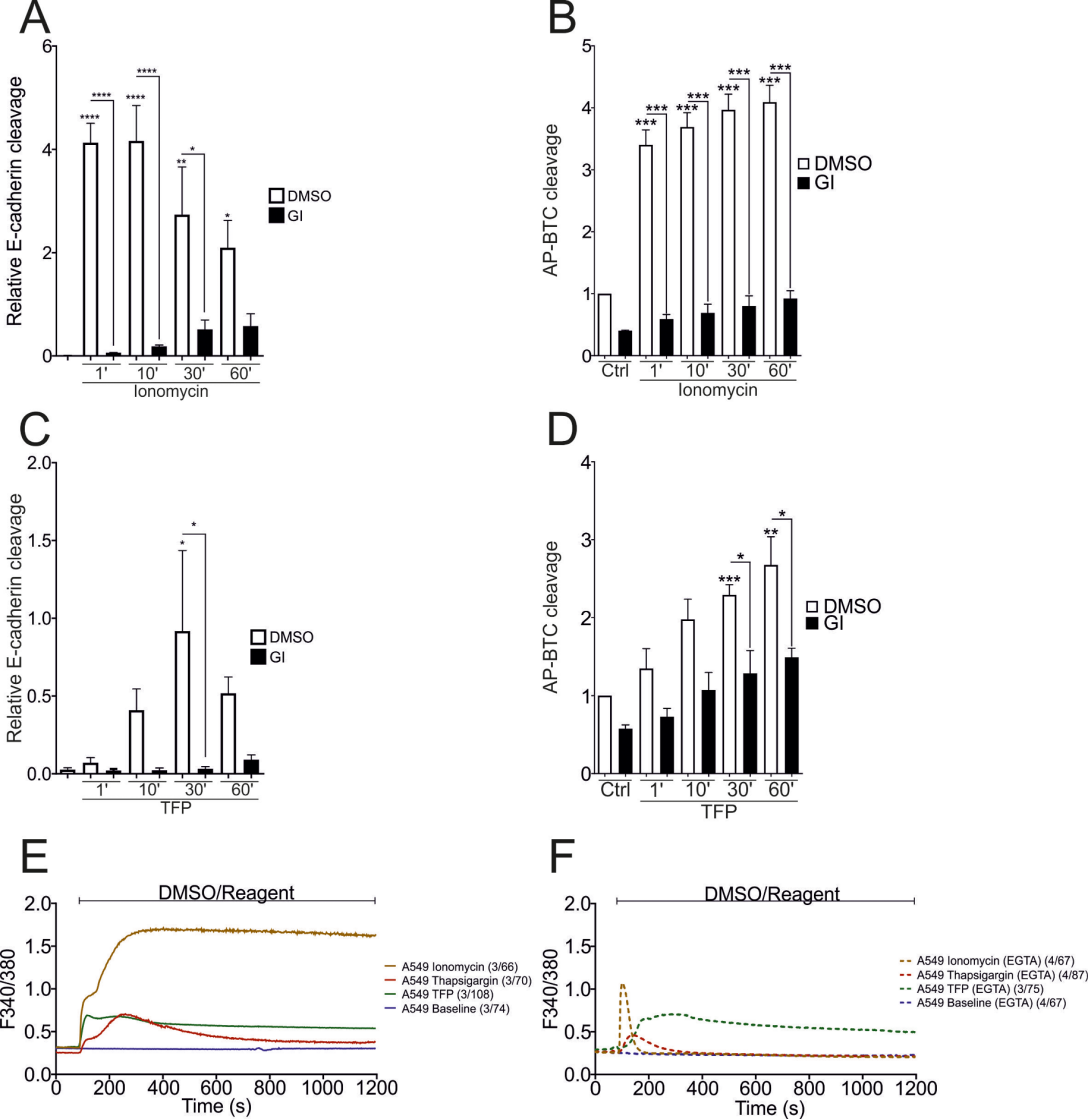
Time-dependence of Ca^2+^-induced ADAM10 activation. **A**-**D**: Confluent A549 monolayers were starved overnight and stimulated with 10 µM ionomycin or 100 µM TFP in the presence or absence of 10 µM GI for different durations (1’=1 min). DMSO (0.1%) served as a vehicle control. Lysates were collected and subjected to E-cadherin cleavage analyses in A and C (*n* = 3). In B and D, cells were transfected with AP-BTC prior to seeding, and AP-BTC cleavage was analyzed via the AP assay (*n* = 3). E, F: A549 cells were grown to 70% confluence on poly-L-lysine coated cover slips, and subjected to calcium imaging. Ionomycin (10 µM), 1 µM thapsigargin and 100 µM trifluoperazine, were automatically injected after 1.5 min of baseline measurement in the presence or absence (Ca^2+^ sequestration with EGTA) of Ca^2+^ in Ringer’s solution, followed by further recording for 20 min. DMSO (0.1%) served as a baseline measurement and vehicle control. The number of experiments across the number of cells N/n is indicated in the figures. The quantitative data are shown as the means + SDs. Asterisks without lines represent differences compared with the control, and those with lines represent differences between groups (* *p* < 0.05, ** *p* < 0.01, *** *p* < 0.001 and **** *p* < 0.0001)

Ionomycin (10 µM) induced maximal cleavage of E-cadherin and AP-BTC in A549 cells within 1 min (1’) after stimulation, which was blocked by ADAM10-inhibition (Fig. 3A and B - SFig. 1 A). Stimulation with TFP (100 µM), however, required 10 min for the significant induction of E-cadherin- (Fig. 3C - SFig. 1B) and betacellulin-cleavage (Fig. 3D). Interestingly, longer incubation times resulted in reduced E-cadherin cleavage upon both ionomycin and TFP stimulation(Fig. 3A, C). Calcium imaging experiments using Fura2-AM were performed to analyze the observed differences in more detail. Ionomycin (10 µM) led to a biphasic (characteristic of internal release from stores and subsequent influx from the extracellular space), rapid and constant increase in intracellular Ca^2+^ levels (Fig. 3E), which was abolished by EGTA treatment, with an only remaining transient increase of Ca^2+^ levels as a product of efflux from internal stores (Fig. 3F - SFig. 1 C). Thapsigargin (1 µM) caused a typical transient increase of intracellular Ca^2+^ levels but did not significantly affect the area under the curve (AUC) or peak amplitude, although a slight decrease was observed in the presence of EGTA (Fig. 3E - SFig. 1 C, D). For TFP (100 µM), a similar peak amplitude was observed which was 3.4-fold lower than that observed for 10 µM ionomycin. Importantly, TFP led to a constant and significant increase in intracellular Ca^2+^ levels, as shown in the AUC, which was not affected by EGTA (Fig. 3E and F - SFig. 1 C, D).

**Fig. 4:**
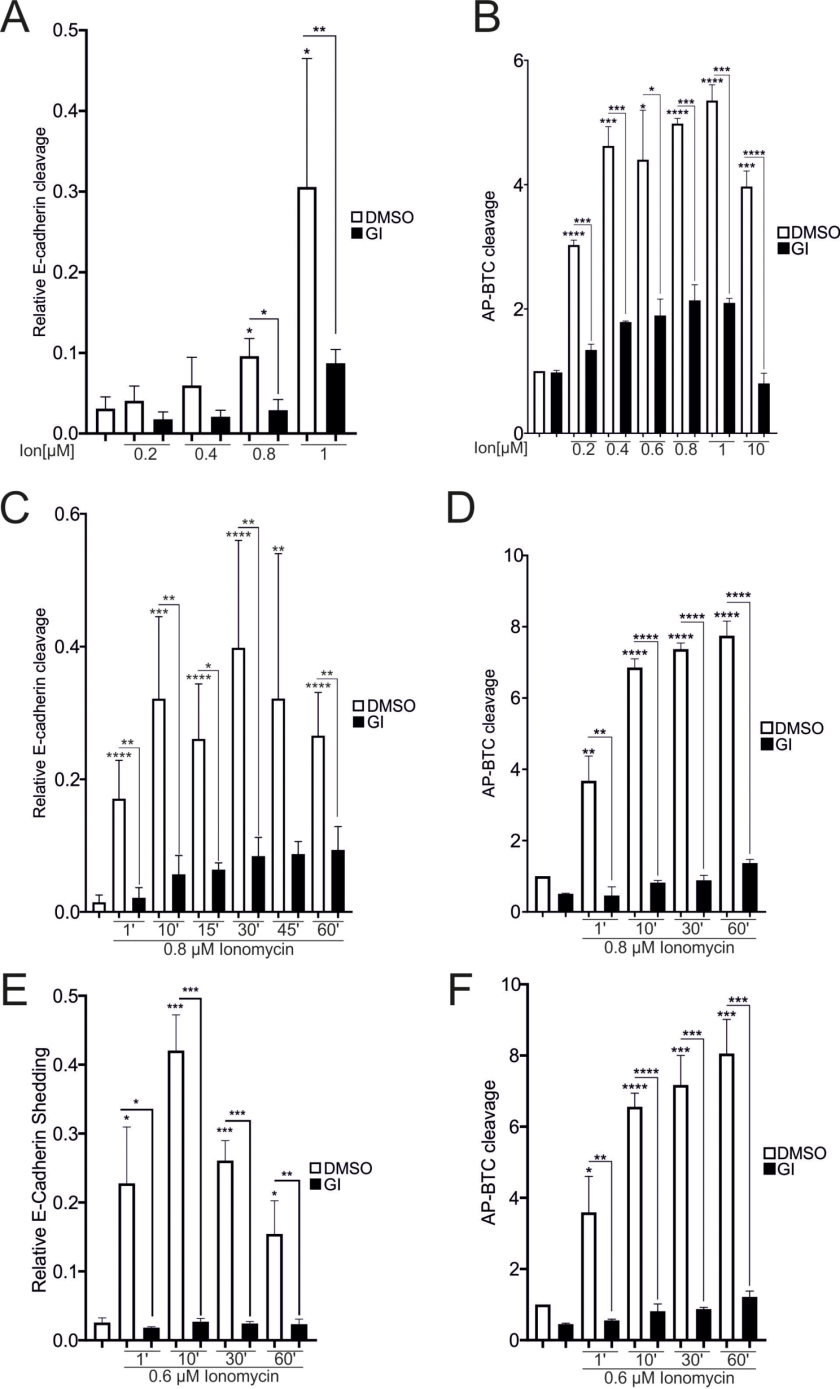
Threshold-dependence of Ca^2+^-induced ADAM10 activation. **A**-**F**: Confluent A549 monolayers were starved overnight and stimulated with different concentrations of ionomycin in the presence or absence of 10 µM GI for 30 min (**A**, **B**) or at different time points (**C**-**F**). DMSO (0.1%) served as a vehicle control. Lysates were collected and subjected to E-cadherin cleavage analysis in A, C, and E (*n* = 3). In B, D, and F, the cells were transfected with AP-BTC prior to seeding, and AP-BTC cleavage was analyzed via the AP assay (*n* = 3). Asterisks without lines represent differences compared with the control, and those with lines represent differences between groups (* *p* < 0.05, ** *p* < 0.01, *** *p* < 0.001 and **** *p* < 0.0001)

Thus, a feasible interpretation is that either a certain threshold or temporal pattern needs to be reached for the Ca^2+^-dependent activation of ADAM10. Considering this information and the potential cytotoxic effects of 10 µM ionomycin which have been observed in some cases [25], an ionomycin calibration assay was performed. Lower concentrations resulted in less cleavage of both E-cadherin and AP-BTC after 30 min of stimulation (Fig. 4A and B - SFig. 1 A). In terms of the dependence on extracellular calcium levels, AP-BTC cleavage occurred upon stimulation with 0.2 µM ionomycin, whereas significant E-cadherin cleavage was only observed after treatment with 0.8 µM ionomycin (Figs. 2 and 4A and B - Suppl. Figure 2 C). The activity measurements were repeated with two different ionomycin concentrations to investigate whether the activation of ADAM10 only depends on the threshold or also on time: stimulation with 0.8 µM ionomycin as the concentration with a maximal activation of AP-BTC cleavage and a significant increase in E-cadherin cleavage (75% maximal increase), and 0.6 µM as the average concentration between cleavage (0.8 µM) and lack of cleavage (0.4 µM). In contrast to full activation after 1 min with 1 to 10 µM ionomycin (Fig. 3A, B), treatment with 0.8 µM ionomycin for 10 to 15 min was required to reach this level for E-cadherin and even for 30 min for AP-BTC (Fig. 4C and D - Suppl. Figure 1B). Similar results were obtained for stimulation with 0.6 µM ionomycin (Fig. 4E, F).

**Fig. 5:**
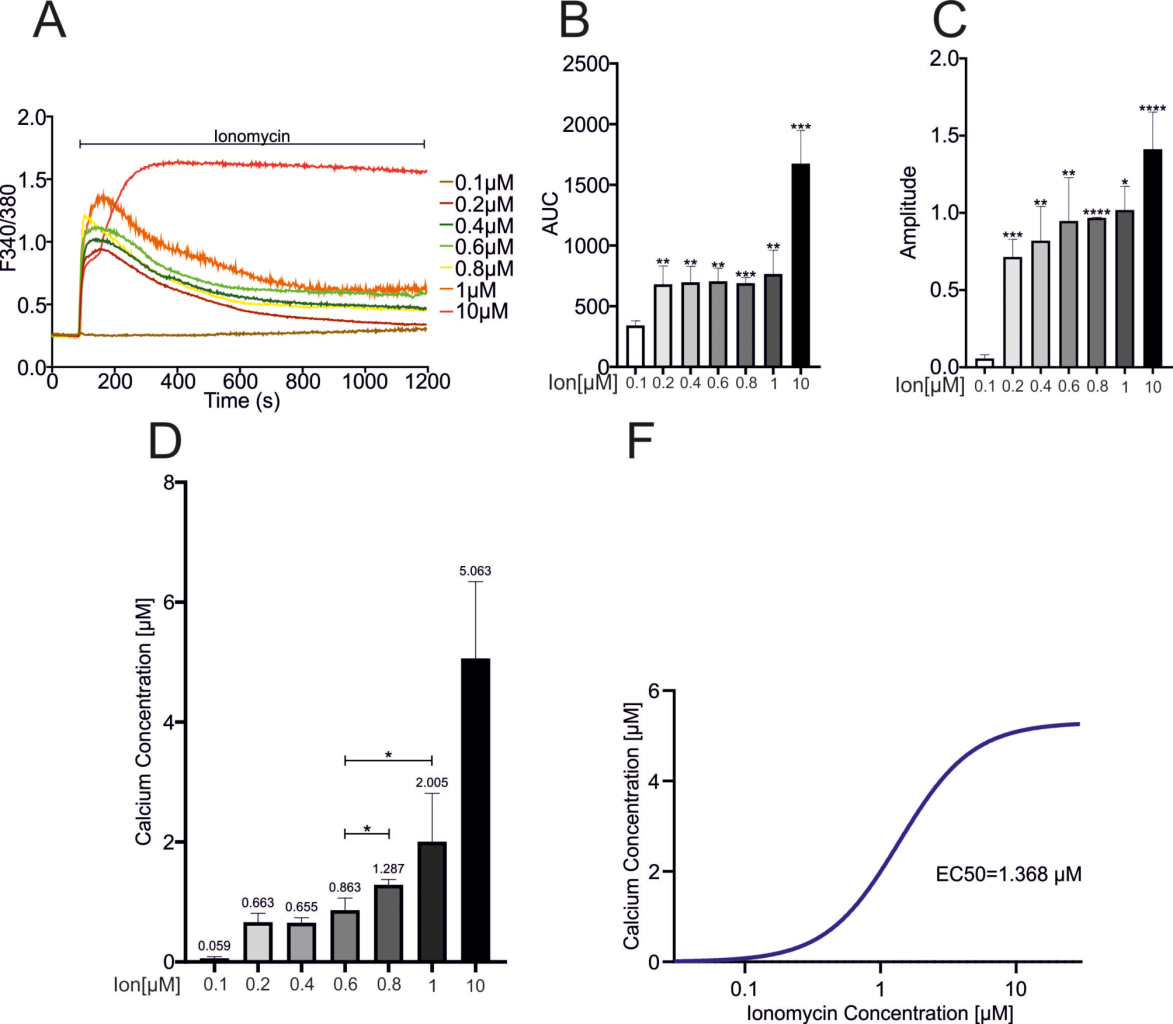
Calculation of the Ca^2+^ concentration threshold. **A**-**C**: A549 cells were grown to 70% confluence on poly-L-lysine coated cover slips and subjected to calcium imaging. After 1.5 min of baseline measurement, increasing concentrations of ionomycin were applied (**A**; for details please refer to the figure notations). Amplitudes (**B**) and areas under the curve (**C**) were quantified and plotted. Data for 10 µM ionomycin were obtained from the results shown in Fig. 3E. The number of experiments/cells used were as follows: 0.1 µM (4/39), 0.2 µM (3/94), 0.4 µM (3/100), 0.6 µM (3/101), 0.8 µM (3/85) and 1 µM (3/92). **D**: The peak values obtained in A were used to calculate the Ca^2+^ concentration. E: A dose-response curve was built from the data obtained in D. Quantitative data are shown as the means + SDs. Statistical analysis was performed using ANOVA plus Tukey´s post hoc test for comparisons between groups. Asterisks without lines represent differences compared with 0.1 µM ionomycin, and those with lines represent differences between groups (* *p* < 0.05, ** *p* < 0.01, *** *p* < 0.001 and **** *p* < 0.0001)

Intracellular Ca^2+^ measurements were performed to determine the threshold of the intracellular Ca^2+^ concentration required for the activation of ADAM10. These data revealed a dose-dependent increase in the peak amplitude and AUC of ionomycin (Fig. 5A, B, C). These ratios were used to calculate the resulting intracellular Ca^2+^ concentrations under these conditions, with 10 µM ionomycin resulting in maximal extracellular Ca^2+^ influx (Fig. 5D). The dose ‒ response curve indicated an EC50 value of 1.368 µM Ca^2+^ (Fig. 5E). Significant, rapid activation of ADAM10 was observed when the cells were stimulated with 0.8 µM ionomycin (Fig. 4A, C), whereas treatment with 0.4 µM ionomycin only slightly increased E-cadherin cleavage, similar to 0.2 µM ionomycin, which was consistent with the Ca^2+^ concentration measurements. Based on the activation observed with slightly lower concentrations such as 0.6 µM and the observed shift in the intracellular Ca^2+^ concentration at this dose (Fig. 5D), the interpolation from the fitting curve indicated that a Ca^2+^ concentration of 0.838 ± 0.2037 µM would consequently be required to reach the activation threshold (Fig. 5D, E). Thus, the activation of ADAM10 by an increase in intracellular Ca^2+^ levels underlies both time-dependent and threshold-dependent control.

### The surface expression and extracellular release of ADAM10 are differentially regulated by Ca^2+^ transients

The current model of Ca^2+^-dependent activation of ADAM10 includes the binding of Ca^2+^ to CaM, followed by the release of Ca^2+^/CaM from the protease and the subsequent cleavage of ADAM10 by furins at the end of the secretory pathway [26]. Interestingly, the CaM inhibitor TFP and ionomycin as ionophore differed in their activation response (Figs. 2 and 3).

**Fig. 6:**
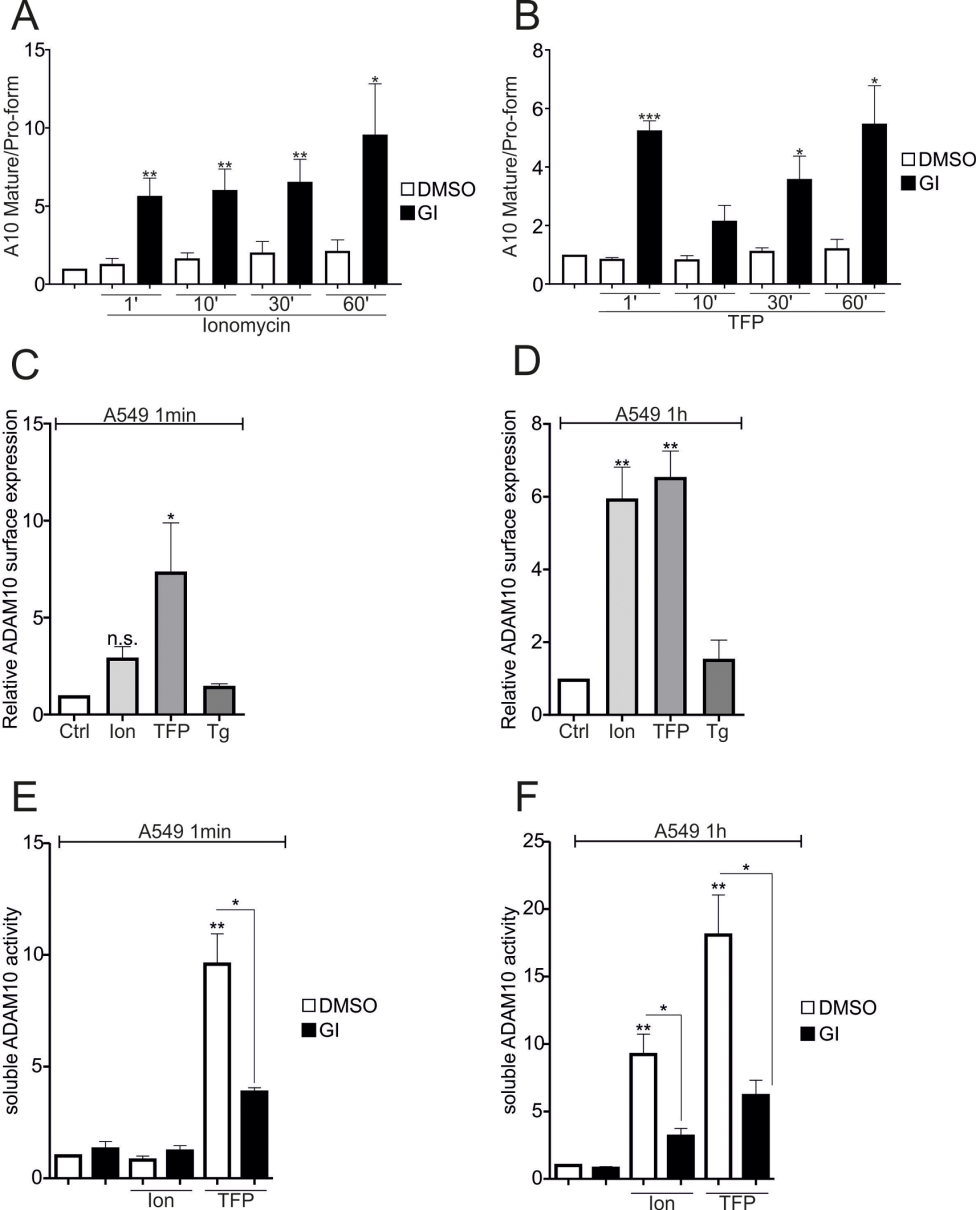
Regulation of cell-associated and soluble ADAM10 activity by Ca^2+^ transients. **A**, **B**: Confluent A549 monolayers were starved overnight and stimulated with 10 µM ionomycin or 100 µM TFP in the presence or absence of 10 µM GI for 1, 10, 30 and 60 min. Lysates were collected and subjected to E-cadherin cleavage analyses. The ratio of mature to pro-form was quantified as measure of maturation by densitometry (*n* = 3). **C**, **D**: Confluent A549 cell monolayers were starved overnight and stimulated with 10 µM ionomycin, 1 µM thapsigargin or 100 µM TFP for 1 min–1 h. Subsequently, the cells were subjected to surface staining for ADAM10, and the fluorescence intensity was measured by flow cytometry (*n* = 4). **E**-**F**. Confluent A549 monolayers were starved overnight and stimulated with 10 µM ionomycin or 100 µM TFP in the presence or absence of 10 µM GI for 1 min–1 h. Supernatants were subjected to FRET-based activity measurements (*n* = 3). In A to F, 0.1% DMSO served as vehicle control. The data are shown as the means + SD. The statistical analyses was performed using a one-sample t-test followed by an FDR analysis. Asterisks without lines represent differences compared with the control, and those with lines represent differences between groups (* *p* < 0.05, ** *p* < 0.01, *** *p* < 0.001 and **** *p* < 0.0001)

Therefore, the maturation and translocation of ADAM10 were investigated in more detail. Reprobing the E-cadherin Western blots with an antibody against ADAM10 revealed that stimulation with 10 µM ionomycin or 100 µM TFP did not affect ADAM10 maturation, as measured by the ratio of the mature form to the pro-form (Fig. 6A and B - SFig. 1 A, B). In all cases, GI increased the cell-associated expression of mature ADAM10 as previously reported [27]. In contrast, an analysis of surface expression revealed the rapid translocation of ADAM10 to the surface upon TFP stimulation (1 min), which was absent in ionomycin stimulated cells (Fig. 6C – SFig. 1 C), indicating that short-term surface expression does not apparently depend on an increase in the intracellular Ca^2+^ concentration. However, longer exposure times (1 h) resulted in a similar increase upon exposure to both treatments (Fig. 6D). However, thapsigargin had no effect. ADAM10 can be released either in exosomes or as a soluble variant [12], resulting in an increase in extracellular activity uncoupled from the plasma membrane. Using a FRET substrate-based approach, we observed a rapid increase in soluble ADAM10 activity upon TFP stimulation, which further increased upon longer exposure (Fig. 6E, F). In contrast, ionomycin required longer exposure times to induce soluble ADAM10 activity, indicating a correlation between ADAM10 surface expression/translocation and soluble ADAM10 activity (Fig. 6C-F). The same experiments were repeated with A431 cells to exclude the possibility of cell-type specific effects, and similar results and kinetics were obtained (Fig. 6 - SFig. 1D-G).

**Fig. 7:**
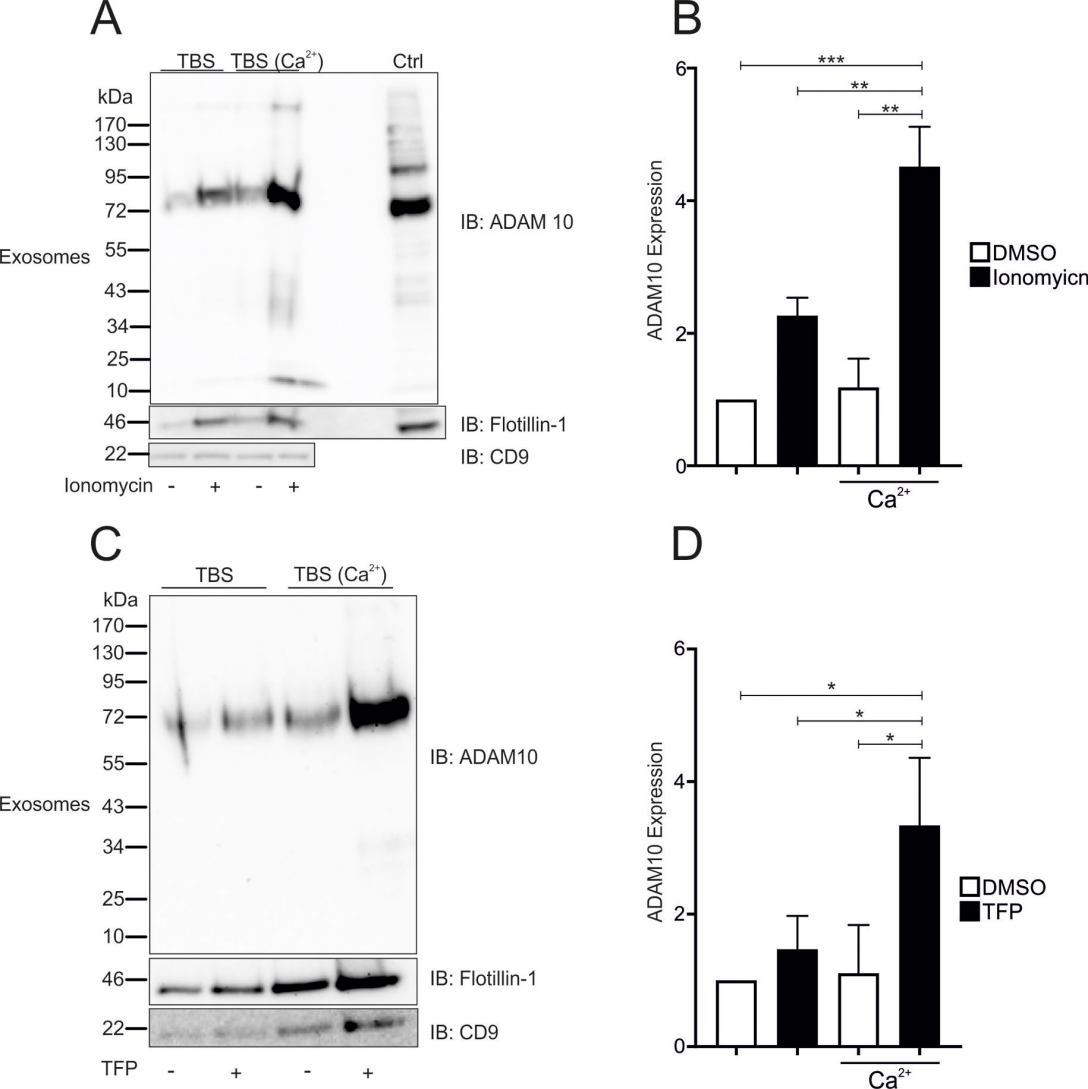
Control of ADAM10 exosomal release by calcium transients. **A**-**D**: Confluent A549 monolayers were starved and stimulated with 10 µM ionomycin (**A**, **B**) or 100 µM TFP (**C**, **D**) in the presence or absence of 2 mM Ca^2+^ in TBS. The supernatant was then subjected to sequential and density centrifugation. The 1.19 g/mL (exosome) fraction was subjected to Western blot analysis by probing with antibodies against ADAM10, flotilin-1 and CD9 (both positive exosome markers). Representative blots are shown in A andC (*n* = 3). The quantitative data are shown as the means + SDs. The statistical analysis was performed using a one-sample t-test followed by an FDR analysis (* *p* < 0.05, ** *p* < 0.01 and *** *p* < 0.001)

Exosome preparations were prepared in the presence or absence of extracellular calcium to distinguish the source of soluble extracellular ADAM10 activity. Nonstimulated cells displayed constant release of ADAM10 in exosomes, which increased upon ionomycin stimulation in the presence but not in the absence of extracellular Ca^2^ (Fig. 7A, B). Interestingly, this increase was associated with an increase in the expression of the exosomal marker CD9, with CD9 being an essential tetraspanin for ADAM10 trafficking and surface localization [28] (Fig. 7 – SFig. 1 A). Similar effects were observed upon stimulation with TFP (Fig. 7C and D -SFig. 7B). However, this increase was not significant.

**Fig. 8:**
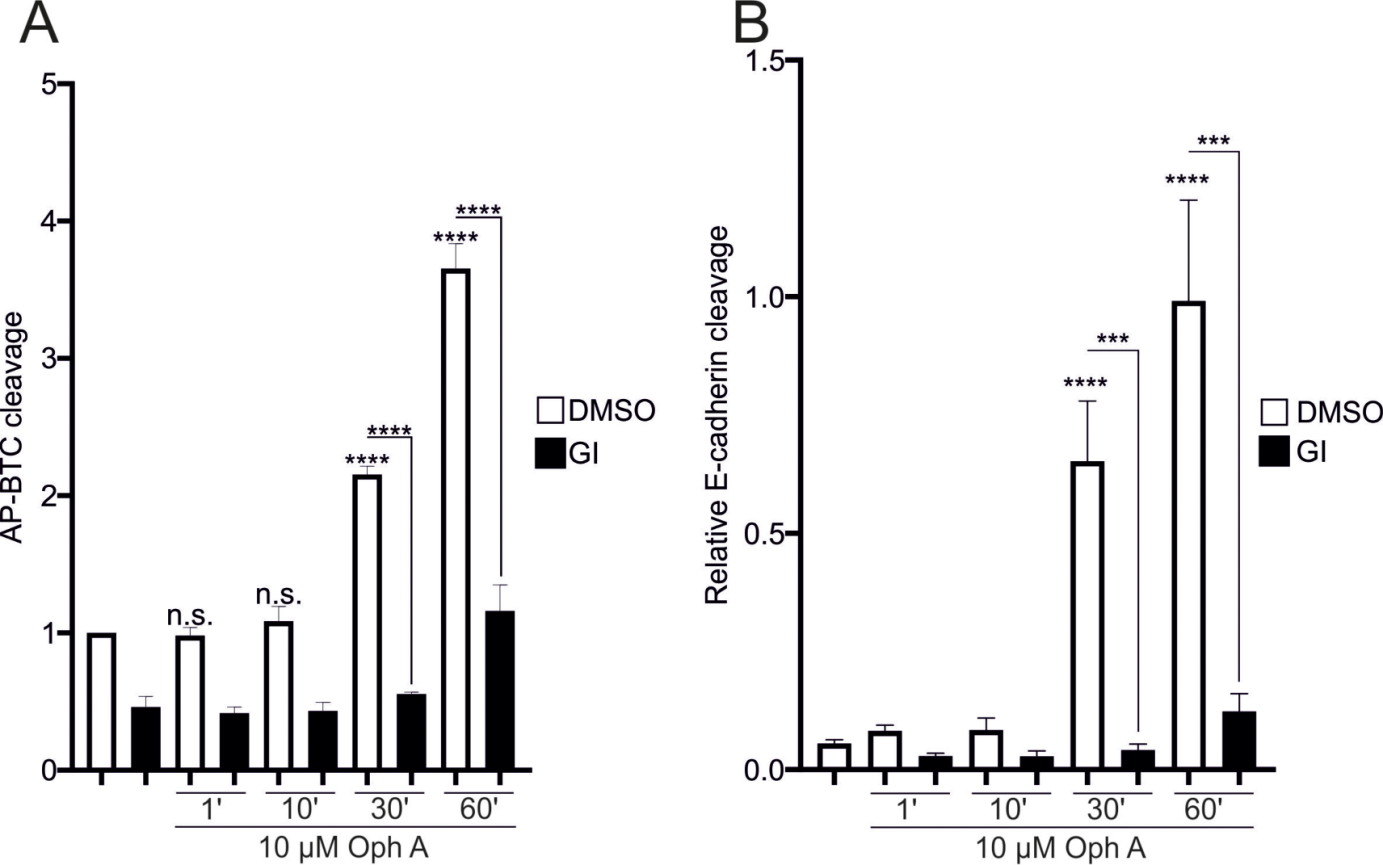
Ophiobolin A-induced ADAM10 activity. **A**, **B**: Confluent A549 monolayers were starved overnight and stimulated with 10 µM ophiobolin A in the absence or presence of 10 µM GI. DMSO (0.1%) served as a vehicle control. In A, cells were transfected with AP-BTC prior to seeding, and AP-BTC cleavage was analyzed via the AP assay (*n* = 3). In B, lysates were collected and subjected to E-cadherin cleavage analyses (*n* = 3). The quantitative data are shown as the means + SDs. Asterisks without lines represent differences compared with the control, and those with lines represent differences between groups (* *p* < 0.05, ** *p* < 0.01, *** *p* < 0.001 and **** *p* < 0.0001)

As mentioned above, TFP acts in a CaM- and Ca^2+^-dependent manner. Experiments were performed with ophiobolin A (OphA), a known CaM inhibitor [29],to analyze the contribution of CaM separately. Compared with TFP stimulation, stimulation with 10 µM OphA resulted in an extended activation time for AP-BTC cleavage of 10 to 30 min (Figs. 3D and 8A). E-cadherin cleavage was obvious after 30 min (Fig. 8B - SFig. 1, A) but was much weaker than that observed upon stimulation with ionomycin (Fig. 3A) or TFP (Fig. 3C). Imaging experiments revealed a slight increase in the level of intracellular calcium, which reached values comparable to those of thapsigargin after longer exposure times and a significantly weaker amplitude (Fig. 8 – SFig. 1, B, C, D). Thus, increased activity increase due to surface translocation or activation on the surface are two processes that are differentially regulated by CaM and intracellular Ca^2+^ levels.

### Spatial control of ADAM10 activation by Ca^2+^ entrance via TRP channels

The ionophore ionomycin is a rather nonspecific stimulus of extracellular calcium entry. Therefore, we questioned whether TRP channels could be involved in the fine-tuning of ADAM10 activation given that growth factor cleavage was recently reported to be a feasible readout for TRP channel activity [10].

**Fig. 9:**
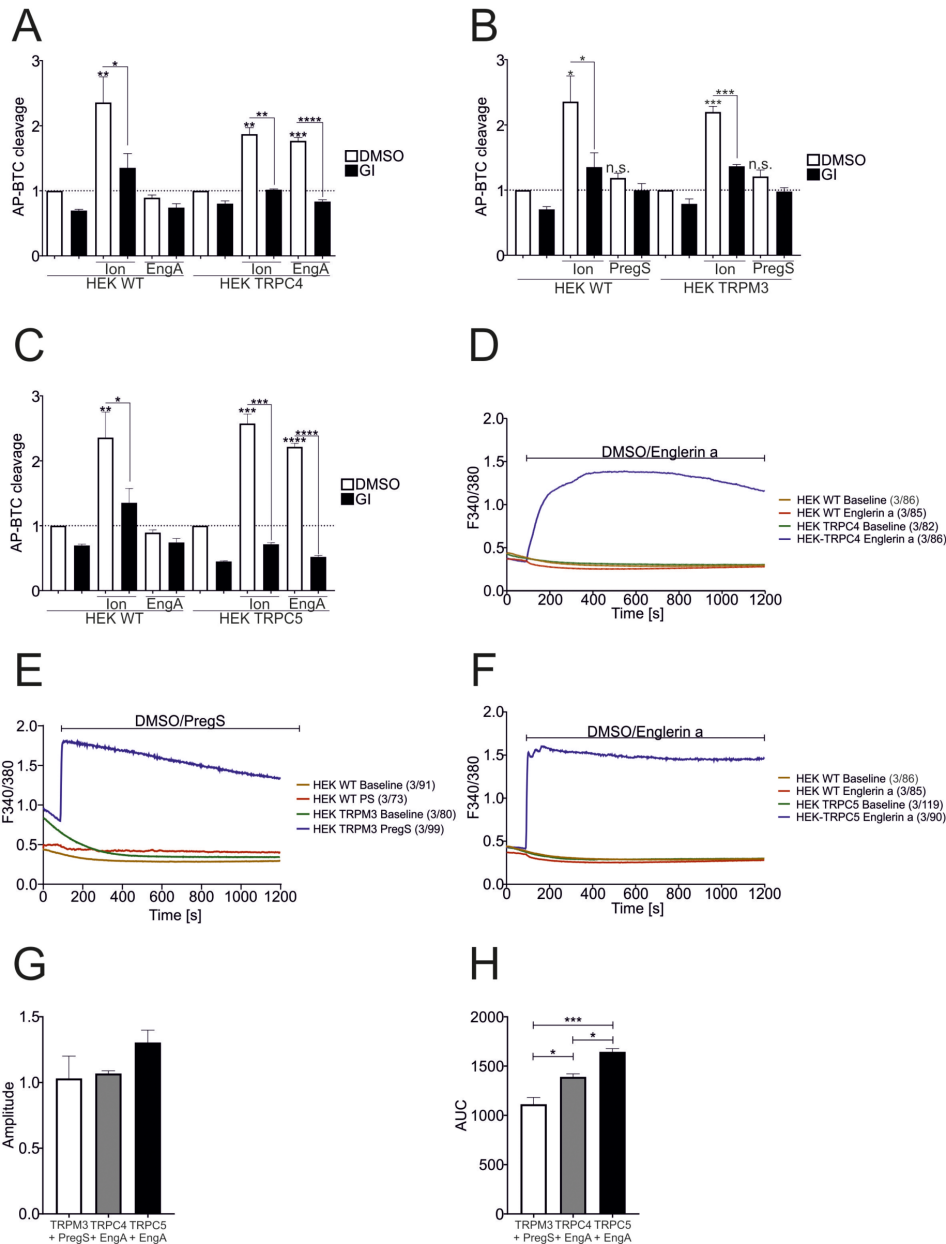
Spatial control of ADAM10 activation by Ca^2+^ influx via TRP channels. **A**-**C**: HEK WT and stable HEK µOR-TRPC4, HEK-TRPM3α2 and HEK-TRPC5 cells were transfected with AP-BTC and stimulated with 10 µM ionomycin, 60 nM Englerin A (EngA) or 100 µM pregnenolone sulfate (PregS) for 30 min in the presence or absence of 10 µM GI. DMSO (0.1%) served as a vehicle control. AP-BTC cleavage was analyzed via the AP assay (*n* = 3). D-H: HEK WT and stable HEK µOR-TRPC4, HEK-TRPM3α2 and HEK-TRPC5 cells were seeded on poly-L-lysine coated cover slips, grown to 70% confluence and subjected to calcium imaging. EngA (60 nM) (**A**, **C**) or 100 µM PregS (**B**) was automatically injected 1.5 min after the baseline measurement, followed by recording for 20 min. DMSO (0.1%) served as the baseline measurement and the vehicle control. The number of experiments across the number of cells N/n is indicated in the figures. The amplitude (**G**) and area under the curve (**H**) were quantified and plotted. The quantitative data are shown as the means + SDs. Statistical analyses were performed using ANOVA followed by Tukey´s post-hoc test for multiple comparisons between groups in G and H. Asterisks without lines represent differences compared with the control, and those with lines represent differences between groups (* *p* < 0.05, ** *p* < 0.01, *** *p* < 0.001 and **** *p* < 0.0001)

Ionomycin led to a general increase in ADAM10 activity in HEK293 cells, which was inhibited by GI (Fig. 9A, B). Stimulation with Englerin A (EngA; 60 nM) [30] activated ADAM10-dependent cleavage of AP-BTC in HEK293 cells overexpressing TRPC4, whereas WT cells did not respond (Fig. 9A). In contrast, pregnenolone sulfate (PregS; 100 µM) a potent activator of TRPM3 [31] did not induce AP-BTC cleavage in TRPM3 overexpressing HEK293 cells (Fig. 9B). Therefore, we questioned whether ADAM10 activation could be restricted to canonical TRP channels. Indeed, stimulation of HEK293 cells overexpressing TRPC5 with 60 nM EngA led to the same induction as that observed for TRPC4-expressing cells (Fig. 9C). One explanation could be differences in the intracellular Ca^2+^ levels upon TRPC4, TRPC5, and TRPM3 activation. Slight differences in the AUC were observed, but the peak amplitude was not affected (Fig. 9G, H). However, all the amplitudes were above the determined threshold of the increase in intracellular calcium levels for ADAM10 activation. Coimmunoprecipitation experiments were performed to investigate whether ADAM10 directly interacts with certain TRP channels. Neither the precipitation of the lysate with the anti-TRP antibody nor with the anti-ADAM10 antibody resulted in co-immunoprecipitation of the respective partner (Fig. 9 – SFig.1 A-F). Thus, ADAM10 activation seems to depend on microdomains formed with certain ion channels and other interacting partners in which the local calcium concentrations might be relatively higher.

## Discussion

In the present study, we observed the time- and threshold-dependent activation of ADAM10. Furthermore, its overall activity, its translocation to the surface and its subsequent release as soluble ectodomains or in exosomes are differentially regulated by calcium transients and calmodulin. Furthermore, we discovered a direct correlation between ADAM10 activation and the opening of canonical TRP channels, identifying novel targeting strategies that need to be investigated in future preclinical and translational studies.

In most of the earlier studies, a fixed concentration of ionomycin (e.g. 1 µM or 10 µM) and a single time point, for ADAM10 activation were used without addressing the time-, concentration- or threshold dependence of Ca^2+^- and CaM-related processes [4, 8, 32, 33]. In COS7 cells, 2.5 µM ionomycin is required to induce ADAM10-dependent AP-BTC cleavage [34]. Furthermore, in previous studies, stimulation times longer than 30 min were used, and rapid activation processes were neglected [4, 7, 35]. We observed the rapid activation of ADAM10 at much lower concentrations (0.6 µM), promoting an increase in the concentration of intracellular Ca^2+^ to 0.838 ± 0.2037 µM. The resting Ca^2+^ concentration in A549 cells was found to be 0.0647 ± 0.0025 µM [36]. This result is consistent with the results obtained in the present study, as 0.1 µM ionomycin did not cause any increase in the intracellular Ca^2+^ concentration, with a calculated Ca^2+^ concentration of 0.059 ± 0.029 µM. Additionally, most of these studies used a single ADAM10 substrate for readout. We used two different ADAM10 substrates to consider substrate-dependent processing and kinetics. Importantly, the alpha-cleavage of E-cadherin by ADAM10 may initiate further processing of the C-terminal fragment by presenilin/γ-secretase [4, 37], resulting in additional CTFs between 17 and 33 kDa. However, these smaller CTFs were not observed under the investigated conditions, even further emphasizing the relevance of ADAM10.

Many studies reporting TFP agonism of ADAM10 address its ability to inhibit CaM [8, 38], simply regulating its translocation to the surface and subsequent activation. However, rapid activation was measured only after stimulation with ionomycin and extracellular calcium influx without translocation to the surface. Ca^2+^ imaging experiments revealed a substantial increase inCa^2+^ levels when ionomycin was applied which was almost ablated when the extracellular Ca^2+^ was removed. On the other hand, TFP led to a stable increase in the Ca^2+^ concentration which seemed to have an intracellular origin because of the increased activity observed in the absence of extracellular calcium. One explanation could be the inhibition of the inhibitory feedback loop of Ca^2+^/CaM acting on IP3R by TFP [39, 40], which would not occur under natural conditions to avoid an overstimulation of the cells. In addition, thapsigargin, which leads to the depletion of internal stores was not able to induce ADAM10 activity. Thus, ADAM10 activation seems to require a certain threshold level, which can be reached over time and is greater than Ca^2+^release from intracellular stores alone, whereas ADAM10 translocation to the surface occurs in a CaM-dependent manner. Furthermore, increased surface translocation alone is not necessarily accompanied by increased activity for which a transient increase in intracellular Ca^2+^ concentrations seems to be needed. Thus, translocation to the surface and Ca^2+^-dependent activation of ADAM10 are partially linked but not causative. Some authors have observed a cytotoxic effect of high concentrations of TFP on A549 cells [41] which could result in increased calcium transients; however, the CaM-dependent effects were confirmed with the CaM inhibitor OphA. The regulation of ADAM10 activity occurs not only through the levels of ADAM10 activation and translocation, but also through its release in exosomes. Reduced ADAM10 and ADAM17 surface expression upon infection and inflammatory stimulation, respectively, are linked to their release in exosomes, which was reduced by Ca^2+^ chelation [12, 42]. However, we observed an increase in both the surface expression of ADAM10 and level of mature ADAM10 in exosomes, indicating that a longer duration of Ca^2+^ transients increases the production and translocation of ADAM10. The tetraspanin CD9 promotes the compartmentalization of ADAM10 [28, 43]. Indeed, we observed an increase in the number of CD9-expressing exosomes upon ionomycin and TFP stimulation, which correlated with an increased presence of mature ADAM10 on these exosomes, suggesting the generation of certain microdomains. After Ca^2+^ related signaling events, Ca^2+^ levels fluctuate, and the intracellular homeostasis of Ca^2+^ levels may be quickly restored through the action of the plasma membrane Ca^2+^ ATPase (PMCA) [44] generating Ca^2+^ microdomains [45]. These Ca^2+^ microdomains, e.g. those generated by the opening of STIM/Orais or TRP channels, could also account for the activation of ADAM10 [46–48]. Indeed, TRP subfamily specific activation of ADAM10 was observed in this study. However, further investigations of other TRP subfamilies might be necessary to examine TRP family specificity in ADAM10 activation. The localization of certain ion channels and ADAM10 in similar membrane microdomains, e.g. upon lipid raft formation under certain conditions [49], adds an additional level to the complex regulation of ADAM10 activity. The involved ion channels and formed microdomains might be cell-type specific. Additional studies will be needed to investigate the localization (e.g. high-resolution microscopy) and composition (e.g. proteomic analysis upon crosslinking) of these microdomains. A global knockout of ADAM10 has proven fatal in mice [19], and global inhibitory approaches are yet to show their effectiveness, since the use of hydroxamate-based compounds is limited by their hepatotoxicity [50]. Other compounds, such as INCB7839 produced mild to moderate adverse events in solid and breast tumors in a recent study [51]. In diverse cancers such as human non-small cell lung cancer and oral squamous cell cancer, TRP channels and ADAM10 have been shown to be overexpressed [52–54]. Increased E-cadherin cleavage and other ADAM10 activities might increase the risk of disease progression in cancer through the promotion of the EMT [6]. Thus, targeting certain Ca^2+^ influx pathways may allow for the development of specific therapeutic options that either increase or decrease the activity of ADAM10 and should be addressed in future translational studies.

## Conclusions

In the present study, we were able to determine the dynamics related to the increase in cytosolic Ca^2+^ levels and CaM inhibition in terms of the kinetics and concentration dependency of the activation of ADAM10 on the cell surface and of the release and activity on exosomes and possibly soluble forms. Additionally, to our knowledge, this study is the first to acknowledge the activation of ADAM10 through particular channels such as the canonical members of the TRP family.

## Electronic supplementary material

Below is the link to the electronic supplementary material.

Supplementary Material 1

Supplementary Material 2

Supplementary Material 3

Supplementary Material 4

Supplementary Material 5

Supplementary Material 6

Supplementary Material 7

Supplementary Material 8

## Data Availability

The datasets used and/or analyzed during the current study are available from the corresponding author upon reasonable request.

## Abbreviations

ADAM: a disintegrin and metalloproteinase
AD: Alzheimer’s disease
AP: Alkaline phosphatase
A10: ADAM10
BTC: Betacellulin
EVs: Extracellular vesicles
GI: GI254023X
TFP: Trifluoroperazine
Ca^2+^: Calcium
CaM: Calmodulin
SERCA: Sarcoplasmic/endoplasmic reticulum Ca^2+^ATPase
CTF: c-terminal fragment
Oph A: Ophiobolin A
Eng A: Englerin A
PregS: Pregnenolone sulfate
