# Supplementary figures and images for: Regulation of ADAM10 activity through microdomain-dependent intracellular calcium changes
Source: Cell Commun Signal. 2024 Nov 4;22:531. doi: 10.1186/s12964-024-01891-5 (PMC11533308; doi:10.1186/s12964-024-01891-5)

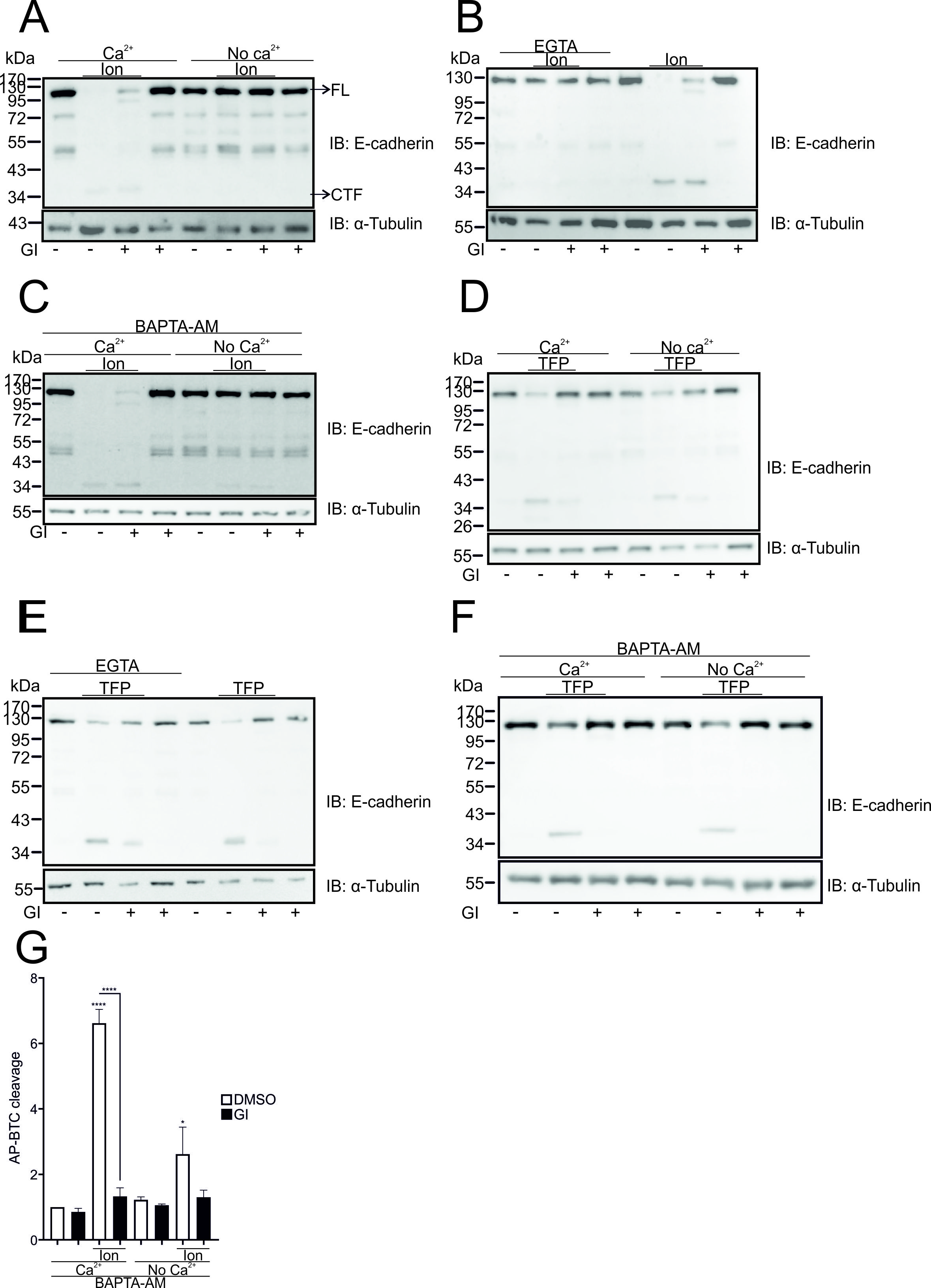

Supplement: Supplementary file 1 — Supplementary Material 1 [file 12964_2024_1891_MOESM1_ESM.jpg]

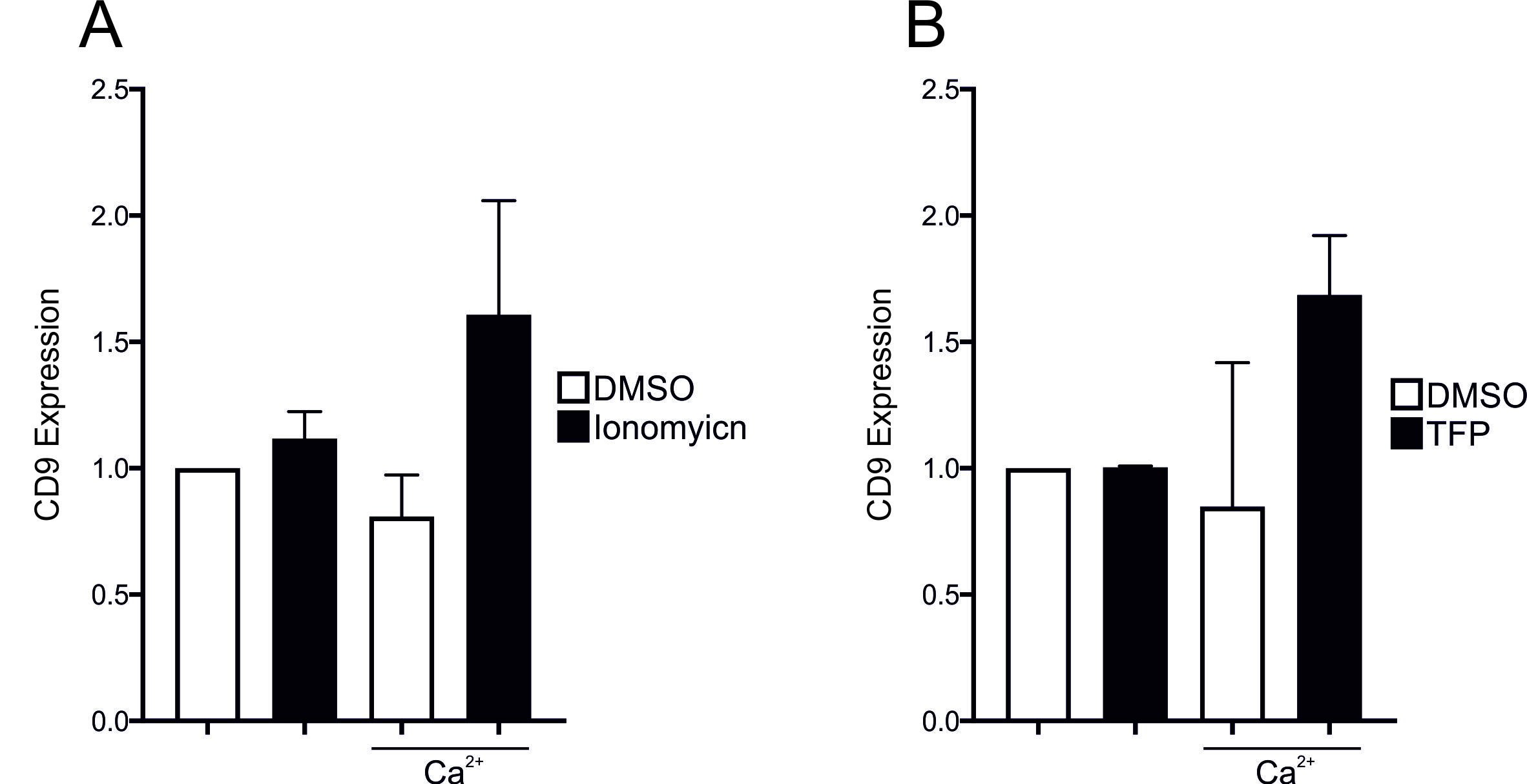

Supplement: Supplementary file 2 — Supplementary Material 2 [file 12964_2024_1891_MOESM2_ESM.jpg]

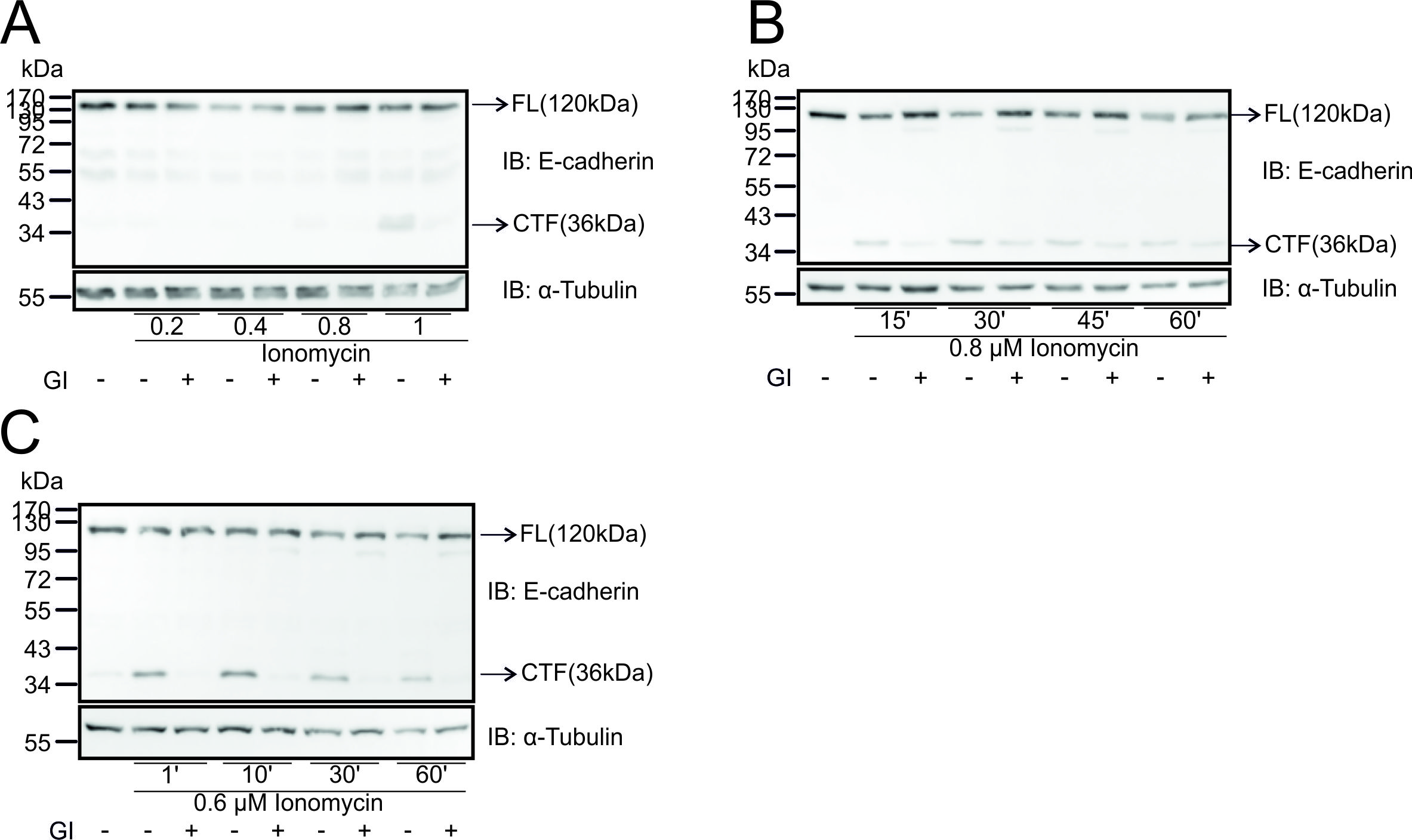

Supplement: Supplementary file 3 — Supplementary Material 3 [file 12964_2024_1891_MOESM3_ESM.jpg]

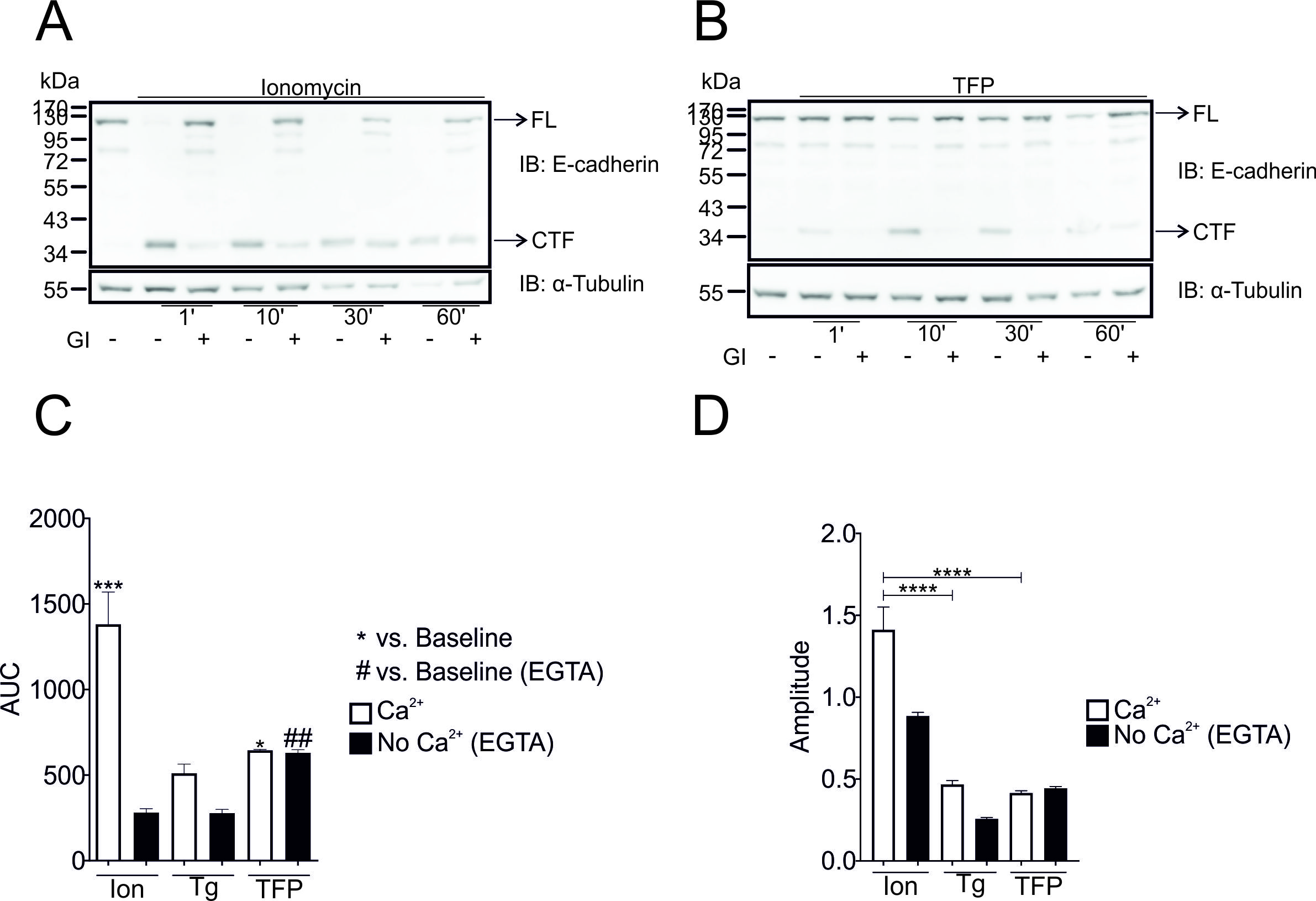

Supplement: Supplementary file 4 — Supplementary Material 4 [file 12964_2024_1891_MOESM4_ESM.jpg]

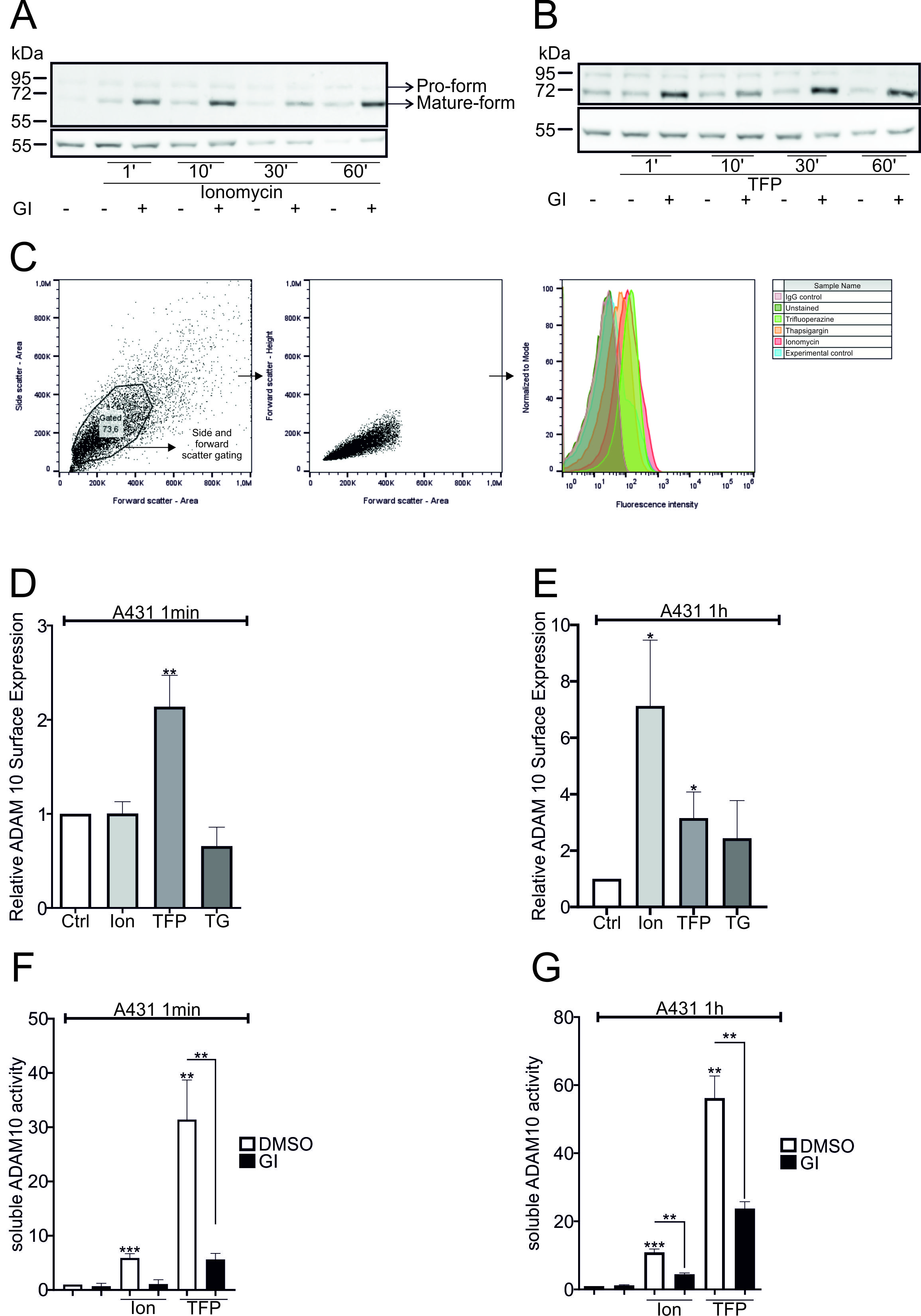

Supplement: Supplementary file 5 — Supplementary Material 5 [file 12964_2024_1891_MOESM5_ESM.jpg]

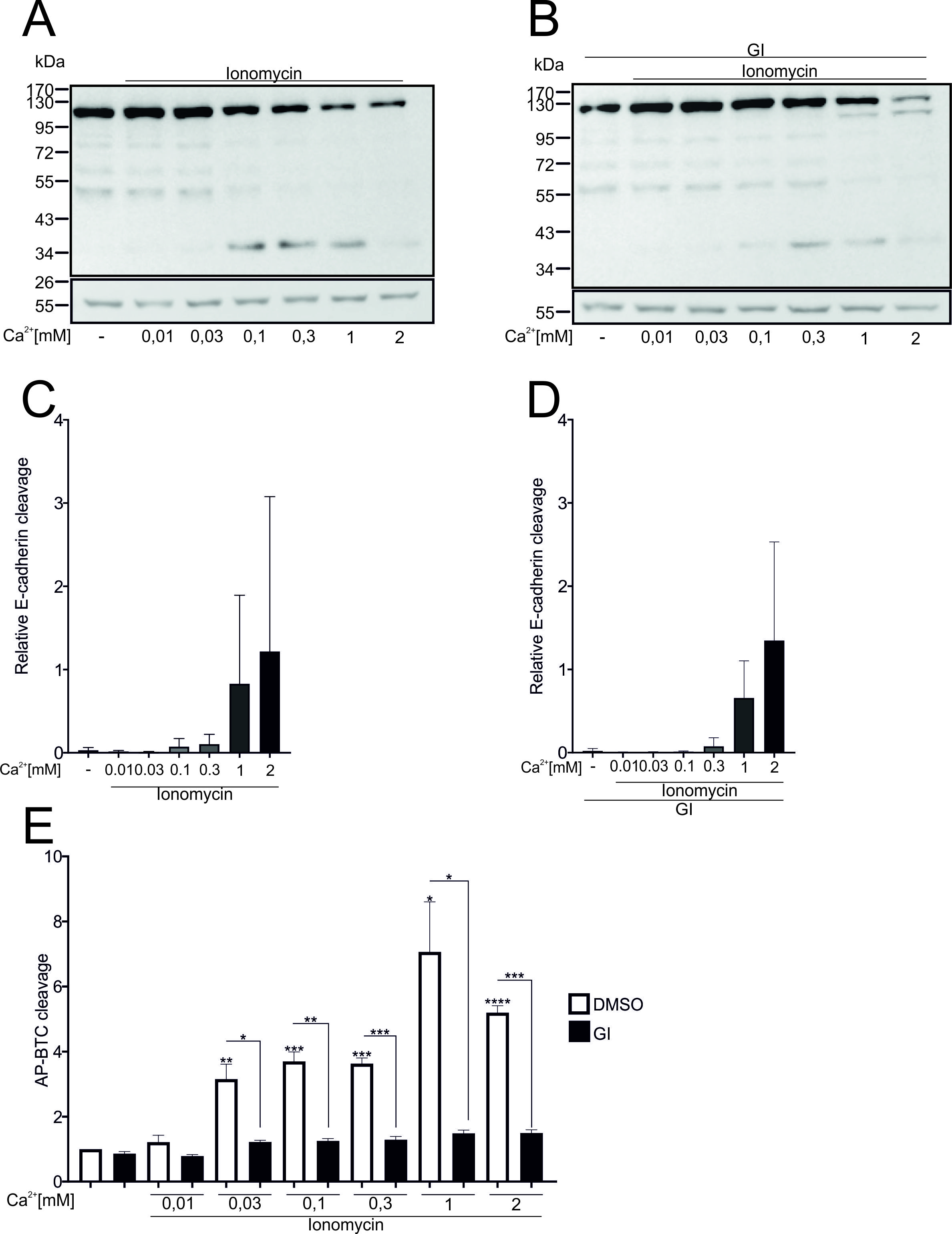

Supplement: Supplementary file 6 — Supplementary Material 6 [file 12964_2024_1891_MOESM6_ESM.jpg]

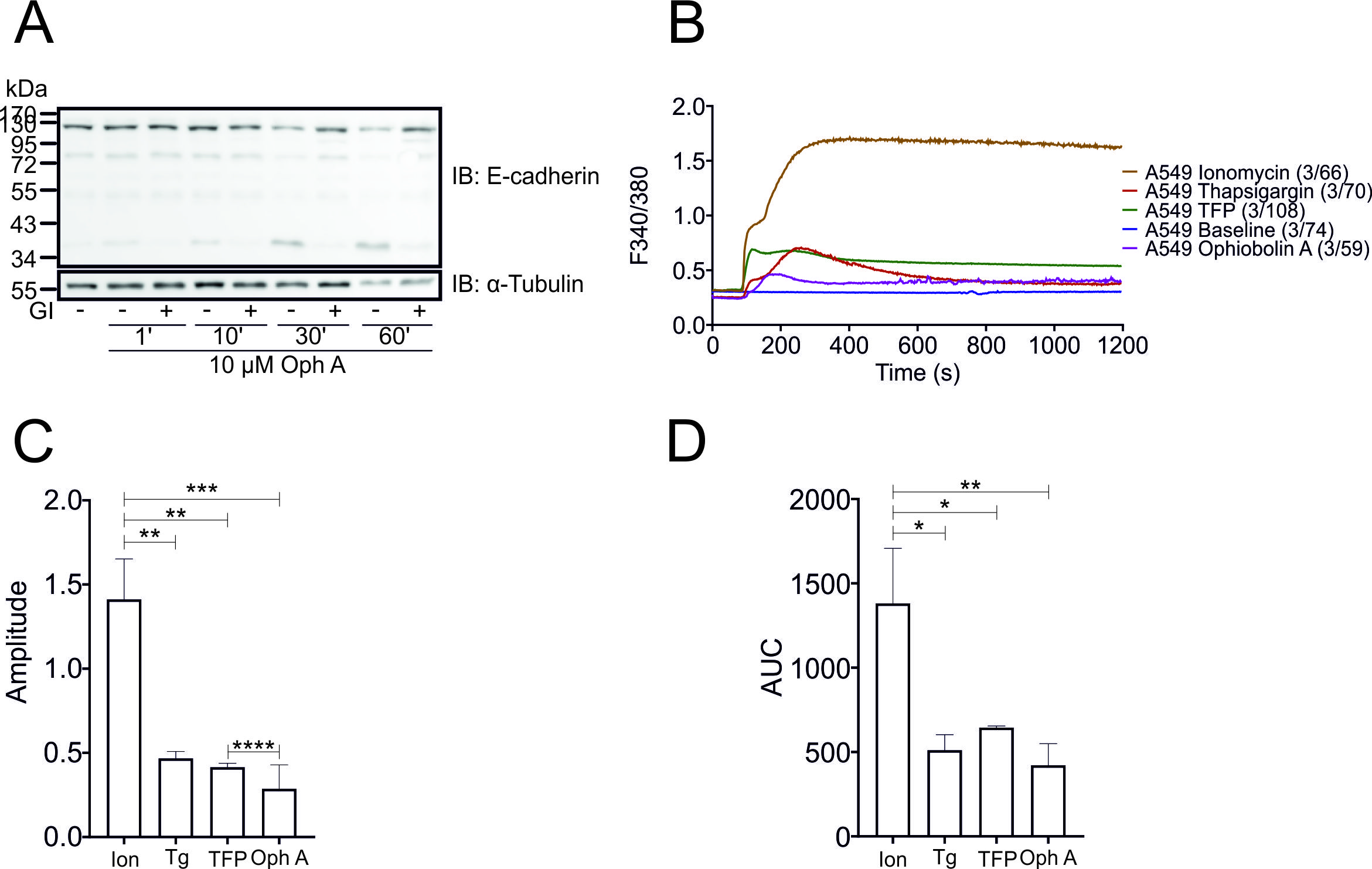

Supplement: Supplementary file 7 — Supplementary Material 7 [file 12964_2024_1891_MOESM7_ESM.jpg]

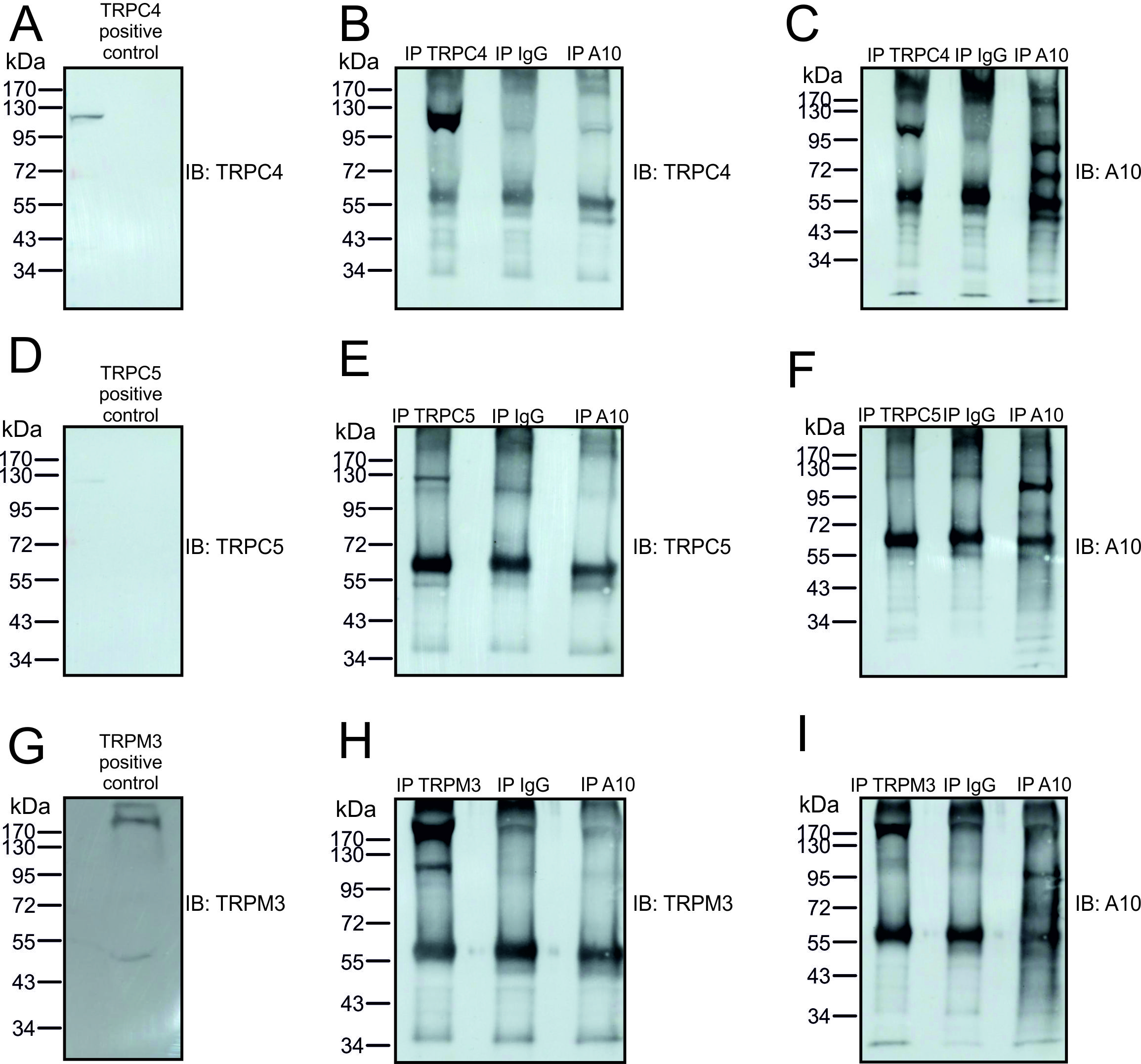

Supplement: Supplementary file 8 — Supplementary Material 8 [file 12964_2024_1891_MOESM8_ESM.jpg]
